# Tumor‐Associated Macrophage‐Derived Exosomal LINC01232 Induces the Immune Escape in Glioma by Decreasing Surface MHC‐I Expression

**DOI:** 10.1002/advs.202207067

**Published:** 2023-04-25

**Authors:** Junjun Li, Keshan Wang, Chao Yang, Kai Zhu, Cheng Jiang, Minjie Wang, Zijie Zhou, Nan Tang, Qiangping Wang, Siqi Wang, Pengwei Shu, Hongliang Yuan, Zhiyong Xiong, Jinsong Li, Tao Liang, Jin Rao, Xuan Wang, Xiaobing Jiang

**Affiliations:** ^1^ Department of Neurosurgery, Union Hospital Tongji Medical College Huazhong University of Science and Technology 1277 Jiefang Avenue Wuhan Hubei 430022 China; ^2^ Department of Urology Union Hospital Tongji Medical College Huazhong University of Science and Technology 1277 Jiefang Avenue Wuhan Hubei 430022 China; ^3^ Tianjin Institute of Industrial Biotechnology Chinese Academy of Sciences Tianjin Tianjin 300308 China; ^4^ Department of Radiology Union Hospital Tongji Medical College Huazhong University of Science and Technology 1277 Jiefang Avenue Wuhan Hubei 430022 China; ^5^ Department of Ultrasound Union Hospital Tongji Medical College Huazhong University of Science and Technology 1277 Jiefang Avenue Wuhan Hubei 430022 China; ^6^ Department of Thoracic Surgery Union Hospital Tongji Medical College Huazhong University of Science and Technology 1277 Jiefang Avenue Wuhan Hubei 430022 China; ^7^ Department of Clinical Laboratory Union Hospital Tongji Medical College Huazhong University of Science and Technology 1277 Jiefang Avenue Wuhan Hubei 430022 China

**Keywords:** TAM, CD8^+^ CTL, glioma, immune escape, LINC01232/E2F2/NBR1/MHC‐I

## Abstract

Tumor‐associated macrophage (TAM) infiltration facilitates glioma malignancy, but the underlying mechanisms remain unclear. Herein, it is reported that TAMs secrete exosomal LINC01232 to induce tumor immune escape. Mechanistically, LINC01232 is found to directly bind E2F2 and promote E2F2 entry into the nucleus; the two synergistically promots the transcription of NBR1. The increase in binding between NBR1 binding and the ubiquitinating MHC‐I protein through the ubiquitin domain causes an increase in the degradation of MHC‐I in autophagolysosomes and a decrease in the expression of MHC‐I on the surface of tumor cells, which in turn led to tumor cell escape from CD8^+^ CTL immune attack. Disruption of E2F2/NBR1/MHC‐I signaling with shRNAs or blockade with the corresponding antibodies largely abolishes the tumor‐supportive effects of LINC01232 and inhibits tumor growth driven by M2‐type macrophages. Importantly, knockdown of LINC01232 enhances the expression of MHC‐I on the surface of tumor cells and improves the response to reinfusion with CD8^+^ T cells. This study reveals the existence of critical molecular crosstalk between TAMs and glioma mediates through the LINC01232/E2F2/NBR1/MHC‐I axis to support malignant tumor growth, indicating that targeting this axis may have therapeutic potential.

## Graphical abstract

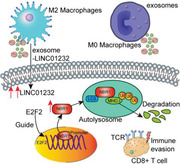

## 1 Introduction

Gliomas are the most aggressive and lethal form of brain tumor and resist conventional therapies, particularly glioblastomas (GBMs).^[^ ^1^ ^]^ The tumor microenvironment plays a critical role in supporting the malignant growth and progression of glioma.^[^ ^2^ ^]^ In gliomas, tumor‐associated macrophages (TAMs) play an important role in supporting tumor growth, as they are an important component of the tumor microenvironment.^[^ ^3^ ^]^ It has been shown that TAM infiltration correlates with glioma progression and tumor grade, and TAM infiltration can be used to predict the survival of glioma patients.^[^ ^4^ ^]^ According to recent studies, TAMs can be functionally classified as tumor‐supportive (M2‐type) macrophages or tumor suppressive (M1‐type) macrophages.^[^ ^5^ ^]^ In contrast to M1‐TAMs, M2‐TAMs generally suppress the immune system and promote tumor malignancy.^[^ ^6^ ^]^ By targeting M2‐TAMs, malignant glioma progression was attenuated in animals, indicating that M2‐TAMs may be useful therapeutic targets for treating glioma.^[^ ^7^ ^]^ Although M2‐TAMs play a critical role in glioma malignancy, their molecular mechanisms remain unknown.

Exosomes are tiny vesicles with phospholipid bilayers that serve as important mediators of intercellular communication.^[^ ^8^ ^]^ Exosomes released by TAMs transfer cargoes such as proteins, ncRNAs, and lipids through the tumor microenvironment, endow cancer cells with different phenotypes, and play important roles in tumor growth, invasion, metastasis, chemoresistance, and angiogenesis.^[^ ^9^ ^]^ Recently, an increasing number of studies have reported that TAM‐derived exosomes may play a role in suppressing immunity by promoting tumor immune escape. Hua Guo et al. reported that M2‐TAMs derived exosomes can reduce the proliferation and cytotoxic activity of CD8^+^ CTLs by inhibiting apoptosis and promoting the progression of glioma, thereby promoting immune escape.^[^ ^10^ ^]^ Bangwei Cao et al. reported that TAM‐derived exosomes mediate the immune escape of gastric cancer cells by promoting PD‐L1 expression in these cells.^[^ ^11^ ^]^ Da Fu et al. reported that TAM‐derived exosomes can inhibit ZC3H12B expression and upregulate IL‐6 by delivering miR‐155‐5p, thereby inducing immune escape and tumorigenesis in patients with colon cancer.^[^ ^12^ ^]^ Qianming Du et al. reported that CC motif chemokine ligand 5 (CCL5) secreted by TAMs promotes colon cancer by inducing the deubiquitination and stability of the PD‐L1 protein in colon cancer cells and inhibiting CD8^+^ CTL‐mediated cell killing to promote cellular immune escape.^[^ ^13^ ^]^ This evidence implies that TAM exosomes play an important role in tumor immune escape.

To explore the role of TAM‐derived exosomes in glioma immune escape, we utilized previously isolated M0/M2‐TAM exosomes in vitro for high‐throughput RNA sequencing. We found that LINC01232 was highly expressed in M2‐TAM‐derived exosomes, and functional experiments confirmed that exosomes‐LINC01232 derived from M2‐TAMs promoted the immune escape of glioma cells by evading CD8^+^ CTL cells. In addition, blocking the synthesis of exosomes or knocking down LINC01232 expression can attenuate the ability of M2‐TAMs to induce glioma immune escape. The lack of MHC class I molecule presentation function is often one of the main reasons for tumor immune escape.^[^ ^14^ ^]^ For example, polycomb repressive complex 2 (PRC2) induces cooperative transcriptional silencing of the MHC‐I antigen processing pathway, thereby promoting T‐cell‐mediated immune escape.^[^ ^15^ ^]^ Alec C Kimmelman et al. reported that the autophagy transport receptor NBR1 binds to MHC‐I through the ubiquitin‐binding domain, mediates the degradation of MHC‐I molecules in autophagolysosomes, and promotes immune escape in patients with pancreatic cancer.^[^ ^16^ ^]^ According to our study, we found that M2‐TAM exosomes, derived from LINC01232, promote tumor immune escape through the E2F2/NBR1/MHC‐I axis. Disruption of LINC01232/E2F2/NBR1/MHC‐I by shRNA or blocking the corresponding antibodies largely diminished the tumor‐supportive effects of LINC01232 and suppressed tumor growth driven by M2‐type macrophages.

## 2 Results

### 2.1 M2‐TAM‐derived Exosomes Promote Glioma Immune Escape

**Figure 1:**
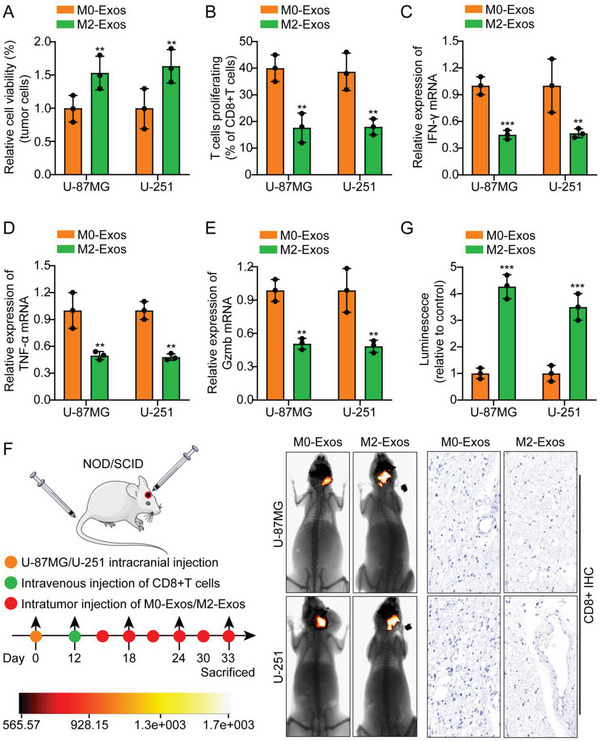
M2‐TAMs‐derived exosomes promote glioma immune escape. A) M2‐Exos significantly inhibited T‐cell‐mediated tumor cell killing compared with that in the presence of M0‐Eoxs. B–E) Flow cytometry and real‐time quantitative PCR results indicated that CD8^+^ T cells cocultured with M2‐Exos‐cultured glioma cells showed lower proliferation and expression of IFN‐*γ*, TNF‐*α*, and Gzmb. F) Schematic diagram of the animal experiments. G) Typical in vivo animal imaging pictures, a bar chart showing typical data, and pictures showing IHC analysis of CD8^+^ expression in different groups. Scale bar, 50 um.The means ± SDs are provided (*n* = 3). ^**^ *p* < 0.01 and ^***^ *p* < 0.001 according to two‐tailed Student's t tests or one‐way ANOVA followed by Dunnett tests for multiple comparisons.

THP1 cells have been widely used as a macrophage model for many studies because primed THP1 cells have several macrophage characteristics.^[^ ^17^ ^]^ To mimic TAMs, we polarized THP1 cells into M2‐TAMs.^[^ ^18^ ^]^ Elevated expression of the M2‐TAM markers CD163, IL‐10, IL1RN, TGFB1, and CCL2 and reduced expression of the M1 marker TNFA were confirmed by qRT‐PCR (Figure S1a, Supporting Information). Importantly, a cell coculture experiment showed that M2‐Exos significantly inhibited T‐cell‐mediated tumor cell killing compared with that of M0‐Eoxs (**Figure** **1**a). Flow cytometry and qRT‒PCR results showed that CD8^+^ T cells cocultured with M2‐Exos‐cultured glioma cells showed lower proliferation and expression of IFN‐*γ*, TNF‐*α*, and Gzmb (Figure 1b–e). In addition, ELISA results showed that CD8^+^ T‐cell supernatants cocultured with M2‐Exos‐cultured glioma cells showed lower levels of IFN‐*γ*, TNF‐*α*, and Gzmb secretion (Figure S1b, Supporting Information). A nude mouse intracranial orthotopic tumor model was established as follows: U‐87MG‐luc/U‐251‐luc cells were orthotopically implanted into the mouse brain. After 12 days, the mice were divided into two groups, and M0/M2‐Exos were injected into the tumor every 3 days. Activated CD8^+^ T cells isolated from healthy human peripheral blood were injected via the tail vein. The results showed that compared with mice injected with M0‐Exos, mice injected with M2‐Exos had a significantly increased tumor volume. IHC analysis of CD8^+^ in animal transplanted tumor specimens indicated that the M2‐Exos group had significantly reduced expression of CD8^+^ compared with that of the M0‐Exos group (Figure 1f,g; Figure S1c, Supporting Information ). As expected, IF of Ki‐67 in the transplanted tumor specimens of animals showed that the M2‐Exos group had enhanced expression of Ki‐67 compared with the mice in the M0‐Exos group (Figure S1c, Supporting Information). In addition, IF of macrophage markers on the transplanted tumor specimens of animals in each group showed that, compared with the control M0‐Exos group, the proportion of F4/80^+^CD206^+^ cells in the M2‐Exos group was significantly increased (Figure S1d, Supporting Information). To further verify the above conclusions, we repeated the above experiments in T98G cells, and the results showed that the above conclusions could be completely reproduced (Figure S12a–f, Supporting Information). These data demonstrate that exosomes derived from M2‐TAMs isolated in vitro significantly promoted glioma cell evasion of CD8+ CTL cell‐mediated antitumor immune responses.

### 2.2 LINC01232 Secreted by M2‐TAMs is Transferred to Glioma Cells

**Figure 2:**
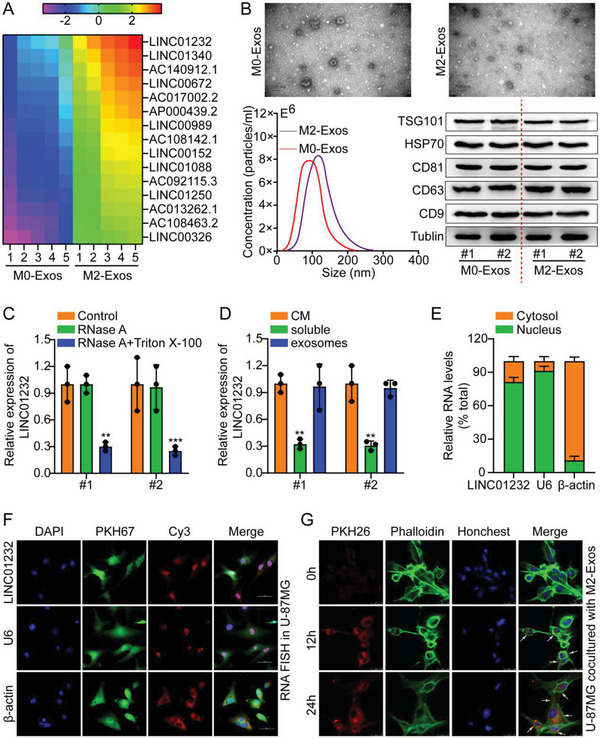
M2‐TAMs‐secreted LINC01232 is transferred to glioma cells A) A heatmap reflecting one of the upregulated lncRNAs identified by RNA sequencing of two pairs of M0‐Exos and M2‐Exos. B) TEM, NTA, and WB were adopted to measure the morphology, size, and surface markers of the exosomes, respectively. Scale bar, 200 nm. C) LINC01232 levels in the CM after M2‐TAM cells were treated with RNase A alone or RNase A and Triton X‐100 simultaneously were measured. D) LINC01232 levels in the CM, soluble fraction, and exosomes were measured. E‐F. RNA FISH analysis of in U‐87MG cells and a bar chart showing typical data. Scale bar, 50 um. G) PKH26‐labeled M2 exosomes were added to phalloidin‐labeled U‐87MG cells. Scale bar, 25 um. The means ± SDs are provided (*n* = 3). ^**^ *p* < 0.01 and ^***^ *p* < 0.001 according to two‐tailed Student's t tests or one‐way ANOVA followed by Dunnett tests for multiple comparisons.

A lncRNA array of M0‐Exos and M2‐Exos was performed to identify exosome‐associated lncRNAs in TAMs. An analysis of hierarchical clustering revealed LINC01232 to be one of the top noncoding RNAs (**Figure** **2**a). Transmission electron microscopy (TEM), nanoparticle tracking analysis (NTA), and Western blotting (WB) were used to measure the morphology, size, and surface markers of exosomes to verify that they were present in the TAM media (Figure 2b). At the same time, we performed WB to detect the purity of the exosome preparations using the exosome negative marker calnexin (Figure S2a, Supporting Information). Our next step was to examine extracellular LINC01232. In the conditioned medium (CM), the level of LINC01232 did not change when M2‐TAMs were treated with RNase A alone; however, after treatment with RNase A and Triton X‐100, the level of LINC01232 in CM decreased (Figure 2c). Additionally, the level of LINC01232 in exosomes was similar to that in the CM (Figure 2d). Based on these findings, exosomes were found to be the primary carriers of extracellular LINC01232.

Subsequently, we measured the LINC01232 content in M0/M2‐TAMs by qRT‒PCR. The results showed a higher level of LINC01232 expression in M2‐TAMs than in primary M0‐TAMs (Figure S2b, Supporting Information). When compared with HA and NHA cell lines, LINC01232 was evidently upregulated in the eight glioma cell lines (Figure S2c, Supporting Information). Knockdown or overexpression of LINC01232 in M2‐TAMs led to upregulation and downregulation of M2‐Exos, respectively (Figure S2d, Supporting Information). Moreover, the subcellular location of LINC01232 was investigated by FISH and subcellular fractionation, and the results showed that LINC01232 localized in both the nucleus and cytoplasm, and mainly in the nuclei of glioma cells (Figure 2e,f). Subsequently, we incubated phalloidin‐labeled U‐87MG cells with PKH26‐labeled M2 exosomes. The IF results showed colocalization of phalloidin lipid dye and PKH26 fluorescence in the incubated U‐87MG cells, demonstrating that exosomes were effectively absorbed by the cells (Figure 2g). Based on these results, LINC01232 appears to be contained in M2‐secreted exosomes and may be capable of being transferred to glioma cells. The level of LINC01232 was determined in U‐87MG and U‐251 cells incubated with exosomes derived from M2/Vector and M2/LINC01232‐OE (M2/1232‐OE). LINC01232 was obviously increased in the cells that were incubated with M2/1232‐OE exosomes (Figure S2e, Supporting Information). After treatment with Annexin V, M2/1232‐OE exosomes failed to increase LINC01232 in U‐87MG and U‐251 cells (Figure S2f, Supporting Information). These results showed that exosomes could transport M2‐secreted LINC01232 to glioma cells.

### 2.3 M2‐TAM‐Derived Exosomal LINC01232 Induces Tumor Immune Escape

**Figure 3:**
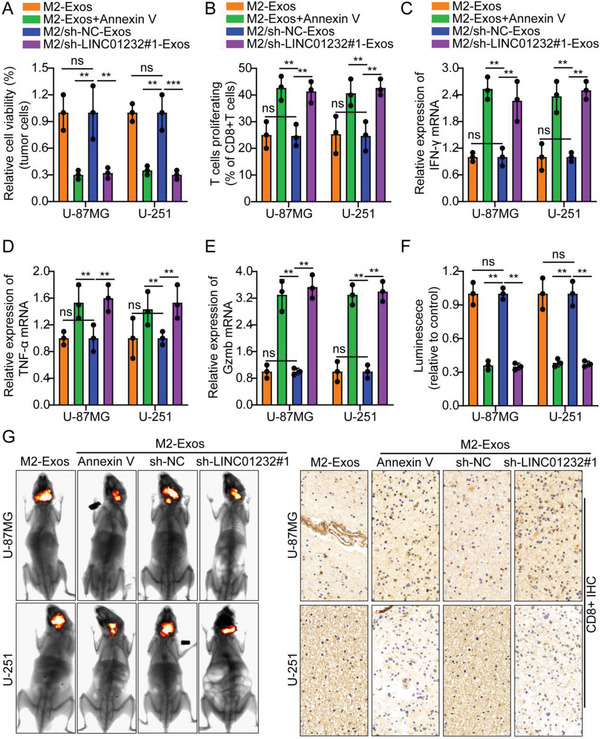
M2‐TAM‐derived exosomal LINC01232 induces tumor immune escape. A) M2‐Exos significantly inhibited T‐cell‐mediated tumor cell killing compared with that in the presence of M0‐Eoxs. However, after adding the exosome inhibitor Annexin V or knocking down LINC01232, the above change was abrogated. B–E) Flow cytometry and real‐time quantitative PCR results indicated that CD8^+^ T cells cocultured with M2‐Exos‐cultured glioma cells showed lower proliferation and expression of IFN‐*γ*, TNF‐*α*, and Gzmb. However, after adding the exosome inhibitor Annexin V or knocking down LINC01232, the above change were abrogated. F,G) Typical in vivo animal imaging pictures, a bar chart showing typical data and pictures following IHC analysis of CD8^+^ expression in the different groups. Scale bar, 50 um. The means ± SDs are provided (*n* = 3). ^**^ *p* < 0.01 and ^***^ *p* < 0.001 according to two‐tailed Student's t tests or one‐way ANOVA followed by Dunnett tests for multiple comparisons. ns, no significant difference.

First, the TCGA database was used to determine the expression level of LINC01232 in glioma and its correlation with patient prognosis. The results showed that LINC01232 was highly expressed in gliomas, and the expression level was inversely proportional to the prognosis of patients (Figure S3a, Supporting Information). We knocked down the LINC01232 gene using lentiviral vectors containing different siRNAs and two shRNAs to understand the biological function of exosomal LINC01232. In our study, shRNA sh‐1232#1 exhibited the strongest suppression of LINC01232 (Figure S3b, Supporting Information). Consistent with the previous result, the cell coculture showed that M2‐Exos significantly inhibited T‐cell‐mediated tumor cell killing compared with M0‐Eoxs. However, after adding the exosome inhibitor Annexin V or knocking down LINC01232, this phenomenon was reversed (**Figure** **3**a). Flow cytometry and real‐time quantitative PCR results indicated that CD8^+^ T cells cocultured with M2‐Exos‐cultured glioma cells had lower proliferation and expression of IFN‐*γ*, TNF‐*α*, and Gzmb; however, after adding the exosome inhibitor Annexin V or knocking down LINC01232, this phenomenon was reversed (Figure 3b–e).

**Figure 4:**
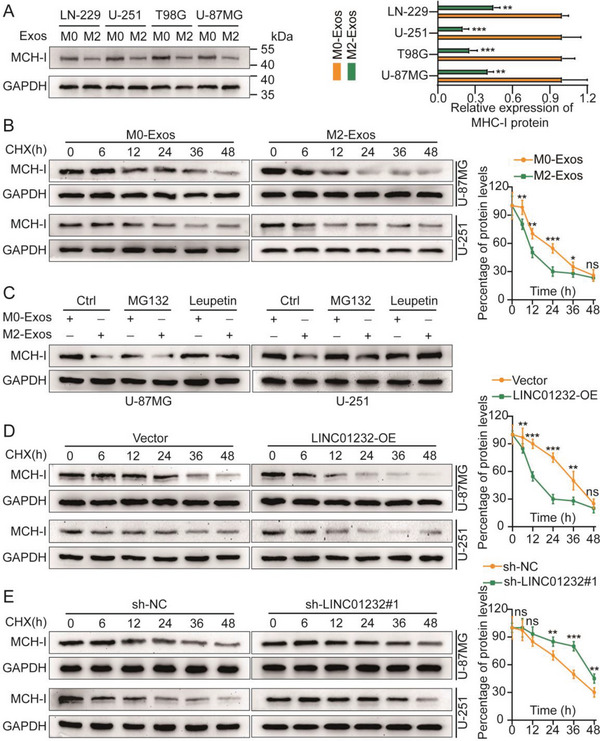
M2‐Exos‐derived LINC01232 mediates MHC‐I expression by regulating the autophagy‐lysosomal pathway. A) Different tumor cell lines were treated with M0‐Exos and M2‐Exos, and the expression level of MHC‐I was detected by WB. The right side shows a bar chart containing typical data. B) U‐87MG and U‐251 cells were treated with M0‐Exos and M2‐Exos, and the expression level of MHC‐I was detected by WB using the CHX assay. The right side shows a typical protein degradation curve. C) U‐87MG and U‐251 cells were simultaneously treated with M0/M2‐Exos simultaneously with the proteasome inhibitor MG132 and the lysosomal inhibitor leupetin, and the expression level of MHC‐I was detected by WB. D,E) LINC01232 was knocked down and overexpressed in U‐87MG and U‐251 cells, respectively, and the expression level of MHC‐I was detected by WB using a CHX assay. The right side shows a typical protein degradation curve. The means ± SDs are provided (*n* = 3). ^*^ *p* < 0.05, ^**^ *p* < 0.01, and ^***^ *p* < 0.001 according to two‐tailed Student's t tests or one‐way ANOVA followed by Dunnett tests for multiple comparisons. ns, no significant difference.

In addition, ELISA results indicated that CD8^+^ T‐cell supernatants cocultured with M2‐Exos‐cultured glioma cells had lower levels of IFN‐*γ*, TNF‐*α*, and Gzmb secretion; however, after adding the exosome inhibitor Annexin V or knocking down LINC01232, this phenomenon was reversed (Figure S3c, Supporting Information). The nude mouse intracranial orthotopic tumor model was established as follows: U‐87MG/U‐251 cells were orthotopically implanted into the mouse brain. After 12 days, the mice were divided into four groups, and M2‐Exos, M2‐Exos+Annexin V, M2/sh‐NC‐Exos, and M2/sh‐1232#1‐Exos were injected into the tumor every 3 days. Activated CD8^+^ T cells isolated from healthy human peripheral blood were injected via the tail vein. The results suggest that compared with mice injected with M2‐Exos or M2/sh‐NC‐Exos alone, M2‐Exos+Annexin V or M2/sh‐1232#1‐Exos significantly decreased the tumor volume. IHC of CD8^+^ in animal transplanted tumor specimens indicated that, compared with mice injected with M2‐Exos or M2/sh‐NC‐Exos alone, the M2‐Exos+Annexin V, or M2/sh‐1232#1‐Exos group had significantly increased expression of CD8^+^ (Figure 3f,g; Figure S3d, Supporting Information). To further verify the above conclusions, we repeated the above experiments in T98G cells, and the results showed that the above conclusions could be completely reproduced (Figure S12g, Supporting Information). Furthermore, IF analysis of Ki‐67 in the transplanted tumor specimens from the animals indicated that the M2‐Exos+Annexin V or M2/sh‐1232#1‐Exos group had lower expression of Ki‐67 than the mice injected with M2‐Exos or M2/sh‐NC‐Exos alone (Figure S3d, Supporting Information). In addition, we also performed IF of macrophage markers on the transplanted tumor specimens of animals in each group, and the results showed that, compared with the control groups M2‐Exos or M2/sh‐NC‐Exos, the proportion of F4/80^+^CD206^+^ in the M2‐Exos+Annexin V or the M2/sh‐1232#1‐Exos group was significantly lower (Figure S4a, Supporting Information). In addition, through TCGA database analysis, we found that the expression of LINC01232 was inversely proportional to the degree of CD8^+^ T‐cell infiltration (FigureS4b, Supporting Information). These experimental results indicated that M2‐Exos‐derived LINC01232 induced tumor immune escape. In contrast, these processes were not promoted by exosomes treated with Annexin V or those with LINC01232 knockdown.

### 2.4 M2‐Exos‐Derived LINC01232 Mediates MHC‐I Expression by Regulating the Autophagy‐Lysosomal Pathway

CD8^+^ T cells typically differentiate into cytotoxic T cells (CTLs) upon activation. Signals that stimulate CTLs for the tumor immune response mainly depend on the formation of the MHC‐I‐antigen peptide‐TCR complex, and defects or changes in MHC‐I molecules on the surface of tumor cells will lead to tumor cells to escape the immune attack by CTLs, thereby conferring a growth advantage to tumor cells.^[^ ^19^ ^]^ Interestingly, our experiments showed that compared with M0‐Exos, M2‐Exos had no significant effect on the mRNA level or the mRNA stability of MHC‐I in glioma cells, but they did significantly reduce the MHC‐I protein level and protein stability (**Figure** **4**a,b; Figure S5a,b, Supporting Information). Therefore, we speculate that M2‐TAM‐derived exosomes promote tumor cells to evade CD8^+^ T‐cell‐mediated antitumor immune responses by promoting the degradation of the MHC‐I protein in glioma cells after translation.

Intracellular proteins are degraded mainly through the following two degradation pathways: the ubiquitin‒proteasome pathway and the autophagy‒lysosome pathway. The ubiquitin‒proteasome pathway is a major pathway that degrades multiple short‐lived proteins with fine specificity and uses temporal control to fine‐tune the steady‐state levels of many regulatory proteins.^[^ ^20^ ^]^ The autophagy‒lysosome pathway mainly degrades cytoplasmic long‐lived proteins and some organelles, contributing to the normal renewal of intracellular components and organelles.^[^ ^21^ ^]^ We treated glioma cells with M0/M2‐Exos, and then simultaneously with the proteasome inhibitor MG132 and the lysosomal inhibitor leupeptin. The results showed that only the lysosomal inhibitor abolished the inhibitory effect of M2‐Exos on the MHC‐I protein (Figure 4c). As expected, in the cycloheximide chase assays (CHX), MHC‐I had a shorter half‐life in cells overexpressing (OE) LINC01232; similarly, in cells downregulating LINC01232, MHC‐I had a longer half‐life (Figure 4d,e). Subsequently, IF experiments were performed using anti‐LAMP1 and anti‐MHC‐I antibodies to observe the effect of MHC‐I expression in lysosomes. The results showed that, relative to the controls, overexpression of LINC01232 attenuated MHC‐I expression in lysosomes, but knockdown of LINC01232 enhanced MHC‐I expression in lysosomes (Figure S5c, Supporting Information). Finally, while overexpressing LINC01232, we treated cells with the autophagy inhibitor chloroquine (CQ) and the lysosomal inhibitor leupeptin and detected MHC‐I protein levels by WB. The results showed that CQ and leupeptin rescued LINC01232 to enhance the degradation of MHC‐I (Figure S6a, Supporting Information). The overexpression of LINC01232 was verified by qRT‒PCR (Figure S6b, Supporting Information). These data suggest that M2‐Exos‐derived LINC01232 mediates the expression of MHC‐I in glioma cells by regulating the autophagy‒lysosome pathway, thereby inducing tumor immune escape.

### 2.5 LINC01232 Binds with E2F2 and Accelerates the Nuclear Translocation of E2F2

**Figure 5:**
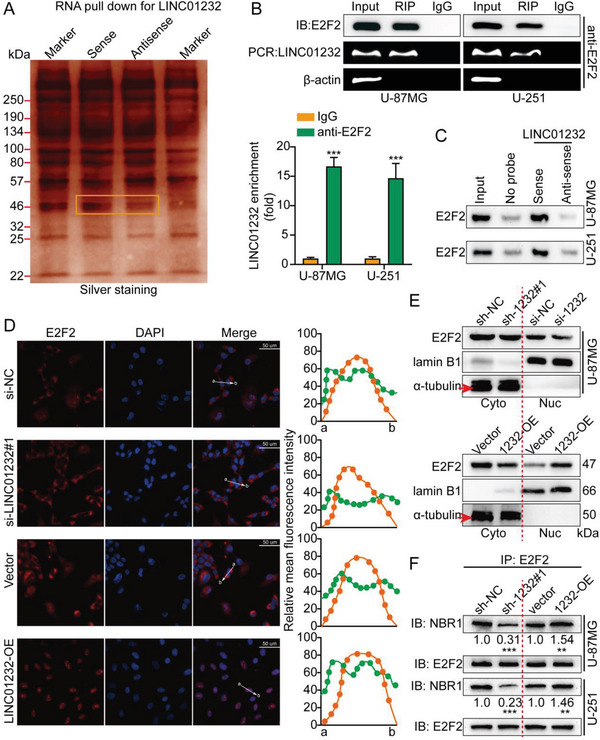
LINC01232 binds with E2F2 and accelerates its nuclear translocation of E2F2. A) The proteins extracted from LINC01232 pull‐down assays were analyzed by MS. B) According to the results of RIP assays with anti‐E2F2 antibodies, E2F2 interacted with LINC01232 in U‐87MG and U‐251 cells. Top, agarose electrophoresis of the PCR product. Bottom, qPCR results following the RIP assay. C) Antisense sequences specific for LINC01232 served as negative controls for WB analysis of the proteins obtained from LINC01232 pull‐down assays. D) Representative IF staining images showing the subcellular E2F2 localization in U‐87MG cells after different treatments were applied are shown. Anti‐E2F2 (red) and DAPI (blue) were used to stain all cells. The average fluorescence intensity of E2F2 and DAPI signals were analyzed using ImageJ. E) WB analysis of nuclear and cytosolic U‐87MG cell lysates was performed after knockdown or overexpression of LINC01232. F) Co‐IP assays with LINC01232 knockdown cells, LINC01232 overexpression cells, and control cells were performed to determine whether E2F2 and NBR1 interact. The means ± SDs are provided (*n* = 3). ^**^ *p* < 0.01 and ^***^ *p* < 0.001 according to two‐tailed Student's t tests.

To explore the specific molecular mechanism by which LINC01232 induces tumor immune escape, RNA pull‐down and mass spectrometry (MS) assays were used to screen for LINC01232‐interacting proteins. E2F2 was identified based on the MS results (Figure S6c, Supporting Information) and RNA pull‐down analysis (**Figure** **5**a). As demonstrated by the RIP assay results, LINC01232 was specifically enriched in E2F2‐immunoprecipitated complexes (Figure 5b). Proteins extracted from LINC01232 pull‐down assays revealed that E2F2 bound specifically bound the sense strand and not to the antisense strand (Figure 5c).

According to the secondary structure predicted using the AnnoLnc databases, we created four truncations of LINC01232 to verify its binding to E2F2. RNA pull‐down assays revealed that the E2F2‐specific binding sequences were located in the 569–1103 nt region of the LINC01232 gene. The interaction domains of E2F2 were identified using RIP assays with Flag‐tagged E2F2 truncations. The A2 domain (172–356) of E2F2 bound to LINC01232 (Figure S6d,e, Supporting Information). In addition, LINC01232 has been shown to regulate the level of E2F2 overall and in the nucleus. Most of the E2F2 was found in the cytoplasm, and a small amount was found in the nuclei of U‐87MG negative control cells. LINC01232 knockdown cells had an almost undetectable level of E2F2 in the nucleus. In contrast, LINC01232‐overexpressing cells had a considerable level of E2F2 in the nucleus (Figure 5d). Compared with the nuclear lysates, cytoplasmic lysates from U‐87MG cells had the most E2F2. As a result of silencing LINC01232, the amount of E2F2 in the nucleus and cytoplasm was significantly reduced in U‐87MG cells; however, the nucleus‐associated E2F2 content was increased following ectopic expression of LINC01232, consistent with our IF results (Figure 5e).

It is well known that the bifunctional transcription factor E2F2 can inhibit or activate gene transcription by binding to promoters. E2F2 has been implicated in glioma, and when it translocates into the nucleus and activates PFKFB4, cancer results.^[^ ^22^ ^]^ In this study, we speculated that LINC01232 could mediate the binding of E2F2 to the promoter region of NBR1. To test this hypothesis, we first used co‐IP to test whether LINC01232 could mediate the mutual binding of E2F2 and NBR1. As expected, overexpression of LINC01232 enhanced this binding, and knockdown weakened this binding (Figure 5f). Additionally, we visualized natural protein complexes using PLA in situ. LINC01232 silencing reduced PLA‐positive protein complexes, including E2F2 and NBR1. Furthermore, the LINC01232 overexpression led to the formation of high‐density clusters of E2F2/NBR1, in agreement with the co‐IP results (Figure S7a, Supporting Information). In addition, we performed a FISH‐IF assay to confirm that LINC01232 directly binds to E2F2 (Figure S7b, Supporting Information). Our experimental results also showed that knockdown and overexpression of LINC01232 had no effect on the levels of E2F2 mRNA and protein (Figure S7c,d, Supporting Information). Taken together, these results showed that LINC01232 could bind with E2F2 and promote its translocation into the nucleus.

### 2.6 LINC01232 Promotes E2F2‐Mediated Transcription of NBR1

**Figure 6:**
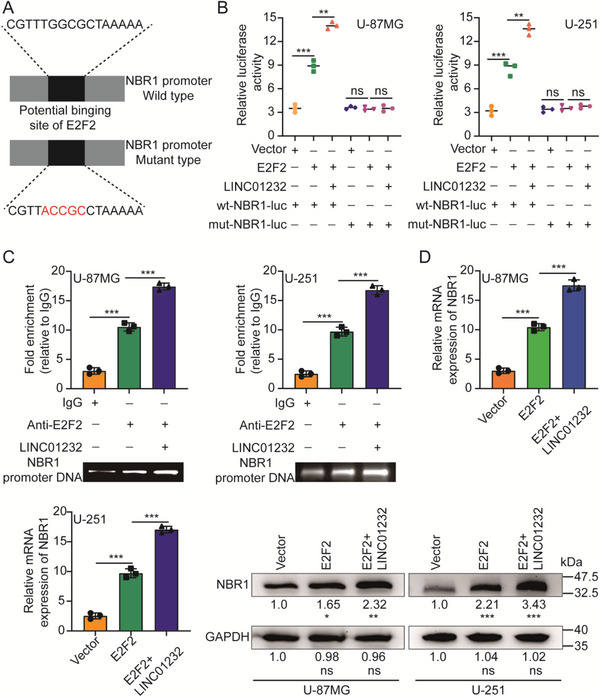
LINC01232 promotes E2F2‐mediated transcription of NBR1. A) We constructed the wild‐type or mutant‐type luciferase vectors based on the potential E2F2‐binding site in the NBR1 promoter. B) Luciferase activity was assayed in U‐87MG and U‐251 cells that were transfected with luciferase vectors (wild type or mutant type) and co‐transfected with expression plasmids (empty vectors, E2F2 expression plasmids, or LINC01232 expression plasmids). C) ChIP analysis of E2F2 (with IgG used as an internal control) was performed, and the coprecipitated DNA was subjected to PCR amplification with primers specific to the NBR1 promoter region. D) The level of NBR1 under ectopic expression of E2F2 or LINC01232 was measured by qRT‐PCR and WB. The means ± SDs are provided (*n* = 3). ^*^ *p* < 0.05, ^**^ *p* < 0.01, and ^***^ *p* < 0.001 according to two‐tailed Student's t tests or one‐way ANOVA followed by Dunnett tests for multiple comparisons. ns, no significant difference.

The expression of target genes is controlled by transcription factors that bind to specific DNA sequences. Using the JASPAR database,^[^ ^23^ ^]^ we found that E2F2 could potentially bind to the NBR1 promoter region. To prove that NBR1 is a transcriptional target of E2F2 and that the regulation can be promoted by LINC01232, luciferase vectors consisting of wild‐type (WT) or mutant (mut) NBR1 promoters were constructed and transfected into U‐87MG and U‐251 cells (**Figure** **6**a). The luciferase assay revealed that overexpression of E2F2 stimulated WT NBR1 promoter activity, as indicated by an increase in luciferase activity, but overexpression had no effect on the activity of the mut‐type NBR1 promoter. Moreover, LINC01232 enhanced the increase in luciferase activity induced by E2F2 (Figure 6b). A ChIP assay was also performed, and the results showed that E2F2 was bound to the NBR1 promoter and that this interaction was enhanced by LINC01232 (Figure 6c). In addition, the enhanced expression of E2F2 significantly upregulated the level of NBR1 mRNA and protein, both of which were further promoted by LINC01232 (Figure 6d). To further verify the above conclusions, we repeated the above experiments in T98G cells, and the above conclusions could be completely reproduced (Figure S13a–c, Supporting Information). Additionally, the RNA pull‐down and RIP assay results did not demonstrate a direct interaction between LINC01232 and NBR1 (Figure S7e,f, Supporting Information). Interestingly, when we mutated the binding site of LINC01232 on E2F2, we found that ΔLINC01232 failed to accelerate the degradation of MHC‐I proteins (Figure S7g, Supporting Information); enhance the binding of E2F2 to NBR1 (Figure S7h, Supporting Information) or promote the nuclear translocation of E2F2 (Figure S8a, Supporting Information). Furthermore, we also found that ΔLINC01232 did not enhance the increase in luciferase activity induced by E2F2 (Figure S8b, Supporting Information), enhance E2F2 binding to the promoter region of NBR1 (Figure S8c, Supporting Information) or potentiate E2F2 upregulation of NBR1 mRNA and protein (Figure S8d, Supporting Information). These results showed that E2F2 directly bound to the promoter region of NBR1 to activate its transcription, and LINC01232 promoted this process.

### 2.7 LINC01232 Promotes Glioma Immune Escape by Regulating NBR1

**Figure 7:**
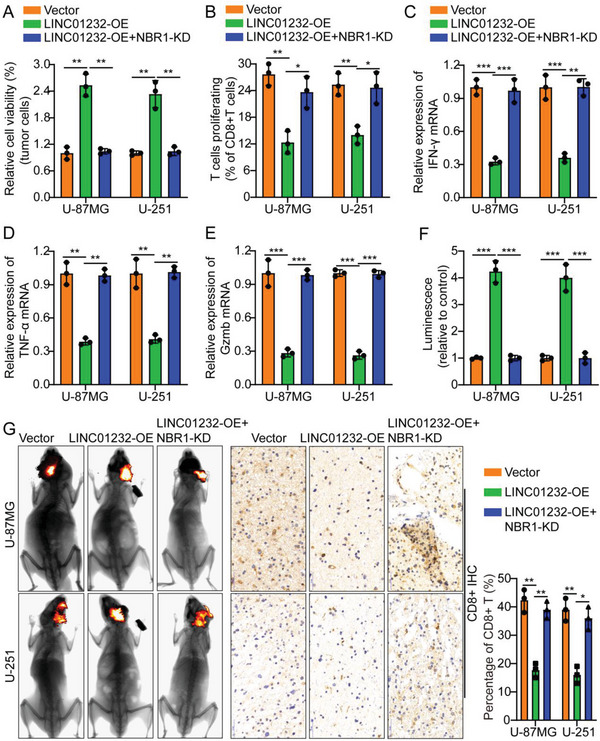
LINC01232 promotes glioma immune escape by regulating NBR1. A) LINC01232‐OE/U‐87MG/U‐251 cells exhibited significantly decreased T‐cell‐mediated tumor cell killing compared with that in cells transfected with the vector; however, NBR1 knockdown and simultaneous overexpression of LINC01232 rescued the above changes. B–E) Flow cytometry and real‐time quantitative PCR results indicated that CD8^+^ T cells cocultured with LINC01232‐OE‐glioma cells showed lower proliferation and expression of IFN‐*γ*, TNF‐*α*, and Gzmb; however, NBR1 knockdown with simultaneous overexpression of LINC01232 rescued the above changes. F,G) Typical in vivo animal imaging pictures, a bar chart showing typical data and pictures following IHC analysis of CD8^+^ expression in the different groups. Scale bar, 50 um. The means ± SDs are provided (*n* = 3). ^*^ *p* < 0.05, ^**^ *p* < 0.01, and ^***^ *p* < 0.001 according to two‐tailed Student's t tests or one‐way ANOVA followed by Dunnett tests for multiple comparisons. ns, no significant difference.

To verify that LINC01232 promotes glioma immune escape by regulating NBR1, we knocked down NBR1 in the glioma cell lines U‐87MG and U‐251 while overexpressing LINC01232. Subsequently, we used WB to verify the knockdown efficiency of NBR1 (Figure S8e, Supporting Information). Interestingly, the results of cell coculture showed that LINC01232‐OE/U‐87MG/U‐251 cells significantly inhibited T‐cell‐mediated tumor cell killing compared with vector; however, knocking down NBR1 while overexpressing LINC01232 could reverse this phenomenon (**Figure** **7**a). Flow cytometry and real‐time quantitative PCR results indicated that CD8^+^ T cells cocultured with LINC01232‐OE glioma cells showed lower proliferation and expression of IFN‐*γ*, TNF‐*α*, and Gzmb; however, knocking down NBR1 while overexpressing LINC01232 could reverse this phenomenon (Figure 7b–e). In addition, ELISA results indicated that CD8^+^ T‐cell supernatants cocultured with LINC01232‐OE glioma cells showed lower levels of IFN‐*γ*, TNF‐*α*, and Gzmb secretion; however, knocking down NBR1 while overexpressing LINC01232 could also reverse this phenomenon (Figure S9a, Supporting Information). A nude mouse intracranial orthotopic tumor model was established as follows: Vector/LINC01232‐OE/LINC01232‐OE+ NBR1‐KD U‐87MG/U‐251 cells were orthotopically implanted into the mouse brain. After 12 days, the activated CD8^+^ T cells isolated from healthy human peripheral blood were injected via the tail vein. The results showed that compared with mice injected with vector, mice in the LINC01232‐OE group had significantly increased tumor volume. The IHC results of CD8^+^ in animal transplanted tumor specimens demonstrated that, compared with mice in the vector group, those in the LINC01232‐OE group had significantly lower expression of CD8^+^; next, NBR1 knockdown with simultaneous LINC01232 overexpression could reverse this phenomenon (Figure 7f,g). The IF results of Ki‐67 in the transplanted tumor specimens from the animals indicated that mice in the LINC01232‐OE group had increased expression of Ki‐67 compared with the mice in the vector group; next, NBR1 knockdown with simultaneous overexpression of LINC01232 could reverse this phenomenon (Figure S9b, Supporting Information). In addition, we overexpressed NBR1 with LINC01232 knockdown, and repeated the above animal experiments. Compared with mice injected with sh‐NC, mice in the sh‐1232#1 group had an obviously decreased tumor volume. The results of IHC analysis of CD8^+^ in tumor specimens transplanted into the animals demonstrated that, compared with mice in the sh‐NC group, those in the sh‐1232#1 group obviously expressed more CD8^+^; next, LINC01232 knockdown with simultaneous overexpression of NBR1 could reverse this phenomenon (Figure S10a, Supporting Information). The results of IF analysis of Ki‐67 in the transplanted tumor specimens from the animals indicated that mice in the sh‐1232#1 group expressed less Ki‐67 than mice in the sh‐NC group; next, LINC01232 knockdown and simultaneous overexpression of NBR1 could reverse this phenomenon (Figure S10a, Supporting Information). As expected, knockdown of LINC01232 promoted the expression of MCH‐I, while overexpression (OE) of LINC01232 inhibited the expression of MCH‐I (Figure S10b, Supporting Information). To further verify the above conclusions, we repeated the above experiments in T98G cells, and the above conclusions could be completely reproduced (Figure S13d, Supporting Information). These results further illustrated that LINC01232 promoted glioma immune escape by regulating NBR1.

### 2.8 Correlation of the LINC01232/E2F2/NBR1/MHC‐I Axis with Clinical Progression

**Figure 8:**
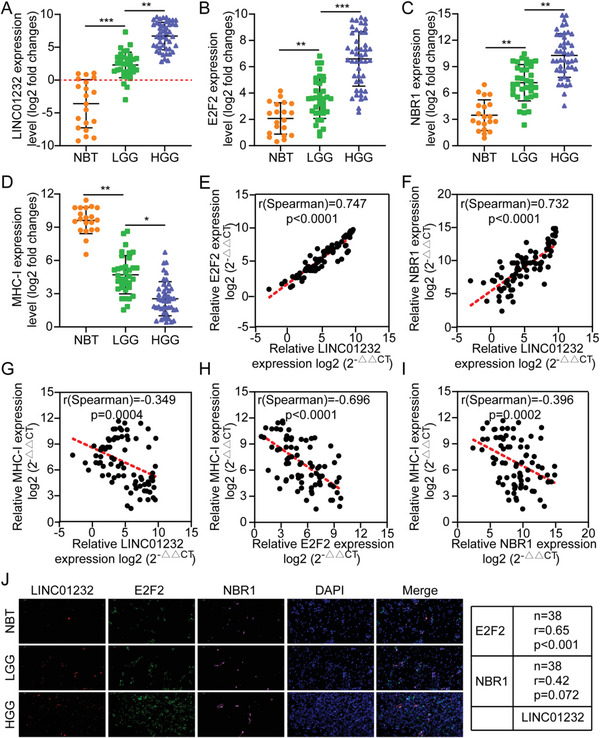
Correlation of the LINC01232/E2F2/NBR1/MHC‐I axis with clinical progression. A–D) Expression levels of LINC01232, E2F2,NBR1, and MHC‐I in NBT, LGG, and HGG determined by qRT‐PCR. E) Spearman correlation analysis between LINC01232 levels and E2F2 levels in tumor tissues from glioma patients. The Pearson's correlation coefficient (r) and *p*‐value are shown in the picture, *n* = 80. The *p*‐value was obtained from Spearman's test. F) Spearman correlation analysis between LINC01232 levels and NBR1 levels in tumor tissues from glioma patients. The Pearson's correlation coefficient (r) and *p*‐value are shown in the picture, *n* = 80. *p*‐value was obtained from Spearman's test. G) Spearman correlation analysis between LINC01232 levels and MHC‐I levels in tumor tissues from glioma patients. The Pearson's correlation coefficient (r) and *p*‐value are shown in the picture, *n* = 80. *p*‐value was obtained from Spearman's test. H) Spearman correlation analysis between E2F2 levels and MHC‐I levels in tumor tissues from glioma patients. The Pearson's correlation coefficient (r) and *p*‐value are shown in the picture showed, *n* = 80. *p*‐value was obtained from Spearman's test. I) Spearman correlation analysis between NBR1 levels and MHC‐I levels in tumor tissues from glioma patients. The Pearson's correlation coefficient (r) and *p*‐value are shown in the picture, *n* = 80. *p*‐value was obtained from Spearman's test. J) Spearman correlation analysis between Linc01060 expression levels and E2F2, NBR1, and MHC‐I expression levels in glioma tissues. The Pearson's correlation coefficient (r) and *p*‐value are shown in the picture; *p*‐value was obtained from Spearman's test. The scale bar represents 50 µm. The means ± SDs are provided (*n* = 3). ^*^ *p* < 0.05, ^**^ *p* < 0.01, and ^***^ *p* < 0.001 according to two‐tailed Student's t tests or one‐way ANOVA followed by Dunnett tests for multiple comparisons. ns, no significant difference.

The LINC01232/E2F2/NBR1/MHC‐I expression levels among normal brain tissues (NBT), low‐grade glioma tissues (LGG), and high‐grade glioma tissues (HGG) were compared. Compared with the expression level in NBT, there was a higher expression of LINC01232/E2F2/NBR1 in tumor tissues, especially in the HGG; however, the expression MHC‐I followed the opposite trend (**Figure** **8**a–d). In addition, through TCGA database analysis, we found that NBR1 and E2F2 are highly expressed in tumor tissues, and the expression of E2F2 was inversely proportional to patient prognosis, consistent with our previous results (Figure S9c,d, Supporting Information). Interestingly, we also found that the expression of E2F2 was inversely proportional to the degree of immune cell CD8^+^ T‐cell infiltration (Figure S11a, Supporting Information). Additionally, the expression levels of the members of the LINC01232/E2F2/NBR1 signaling axis were directly proportional to one another; however, the expression of MHC‐I was inversely proportional to the expression of LINC01232/E2F2/NBR1 (Figure 8e–i). Additionally, FISH, IF, and IHC results showed that the LINC01232 level was positively correlated with E2F2/NBR1 signaling; however, the expression of MHC‐I was inversely proportional to the expression of LINC01232/E2F2/NBR1 (Figure 8j; Figure S11b, Supporting Information)

To assess the pathological and predictive value of LINC01232, ROC analysis was conducted between a LINC01232‐based model, WHO‐based model, and a combination model to predict clinical outcomes. As measured by the area under the curve (AUC), the combination model (0.754) outperformed the WHO‐based model alone (0.612). It seems that the prediction using LINC01232 and WHO stage better predicted clinical outcome than that using WHO stage alone (Figure S11c, Supporting Information). In addition, we examined the correlation between LINC01232 mRNA levels and the clinicopathological characteristics of 80 glioma specimens. Table s1 and Figure S11d (Supporting Information) show the clinical, pathological, and molecular tumor features associated with the LINC01232 mRNA expression level. The results showed that the LINC01232 mRNA expression level was highly associated with the Karnofsky Performance Scale (KPS) score (*p* = 0.001), tumor size (*p* = 0.002), tumor grade (*p* = 0.033), and recurrence (*p* = 0.011). Furthermore, univariate and multivariate Cox regression analyses indicated that a high level of LINC01232 mRNA expression was an independent prognostic factor for poor survival in patients with glioma (Table S2, Supporting Information). Table S2 (Supporting Information) shows that the level of LINC01232 mRNA expression is correlated with tumor grade and tumor recurrence. Taken together, these results demonstrate that LINC01232 expression is positively associated with clinical glioma malignant grade and negatively associated with the prognosis of patients.

Of course, we further determined that the LINC01232/E2F2/NBR1 axis can regulate the expression of MHC‐I through the autophagy‒lysosome pathway. We conducted the following experiments: We knocked down NBR1 in the U‐251 and T98G cell lines and detected the expression level of MCH‐I by WB. NBR1 knockdown promoted the protein expression of MCH‐I. In fact, NBR1 knockdown increased the total and plasma membrane MHC‐I levels in U‐251 and T98G cells (Figure S14a, Supporting Information). Next, we knocked down E2F2 while overexpressing LINC01232 and detected the expression level of MCH‐I by WB. The overexpression of LINC01232 inhibited the protein expression of MCH‐I. As expected, LINC01232 overexpression decreased the total and plasma membrane MHC in‐I level U‐251 and T98G cells. However, E2F2 knockdown with simultaneous overexpression of LINC01232 reversed this phenomenon (Figure S14b, Supporting Information). Next, we knocked down NBR1 while overexpressing LINC01232 and detected the expression level of MCH‐I by WB. The overexpression of LINC01232 inhibited the protein expression of MCH‐I. As expected, LINC01232 overexpression decreased the total and plasma membrane MHC‐I levels in U‐251 and T98G cells. However, NBR1 knockdown with simultaneous overexpression of LINC01232 reversed this phenomenon (Figure S14c, Supporting Information). In addition, we knocked down NBR1 while overexpressing E2F2 and detected the expression level of MCH‐I by WB. The overexpression of E2F2 inhibited the protein expression of MCH‐I. As expected, E2F2 overexpression decreased the total and plasma membrane MHC‐I levels in U‐251 and T98G cells. However, NBR1 knockdown with simultaneous overexpression of E2F2 could reverse this phenomenon (Figure S14d, Supporting Information). The above data again indicated that the LINC01232/E2F2/NBR1 axis can regulate the expression of MHC‐I through the autophagy–lysosome pathway.

## 3 Discussion

**Figure 9:**
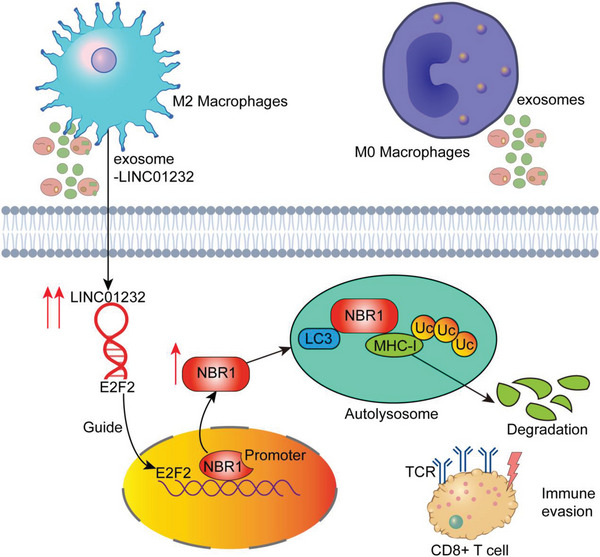
Schematic diagram of the underlying mechanism. T2‐TAMs secrete exosomes rich in LINC01232 into tumor cells, and LINC01232 directly binds E2F2 and promotes E2F2 entry into the nucleus; the two synergistically promote the transcription level of NBR1. The increased binding of NBR1 to the ubiquitinated MHC‐I protein through the ubiquitin domain mediates the increased degradation of MHC‐I in autophagolysosomes, and the decreased expression of MHC‐I on the surface of tumor cells, which in turn leads to tumor cells escape from CD8^+^ CTL immune attack.

Gliomas contain abundant tumor‐supporting TAMs that promote cancer growth,^[^ ^24^ ^]^ and TAM infiltration and poor prognosis in patients with glioma are correlated.^[^ ^4b^ ^]^ As a new and important intercellular communication method, the “cell dialog in microenvironment” mediated by exosomes has piqued the interest of scientists. Recently, a study in Nature reported that adipose tissue exosomes can deliver miRNAs to regulate gene expression in distal tissues.^[^ ^25^ ^]^ Our previous study showed that exosomes derived from glioma stem cells that carry Linc01060 promote glioma progression through the MZF1/c‐Myc/HIF1a signaling axis.^[^ ^26^ ^]^ Herein, we cocultured M2 macrophages with tumor cells and showed that M2 macrophages can promote immune escape from tumors (Figure 1). Therefore, a better understanding of how M2‐TAMs affect glioma maintenance and exert tumor‐promoting effects in the tumor microenvironment is crucial. In this study, we found that TAMs secrete exosomes rich in LINC01232 into tumor cells and that LINC01232 directly binds E2F2 and promotes E2F2 entry into the nucleus; the two synergistically promote the transcription of NBR1. The increase in NBR1 binding to the ubiquitinated protein MHC‐I through the ubiquitin domain caused an increase in the degradation of MHC‐I in autophagolysosomes and a decrease in the expression of MHC‐I on the surface of tumor cells, which in turn led to tumor cell escape from CD8^+^ CTL immune attack (**Figure** **9**).

Macrophage polarization refers to the phenotype of macrophages under given conditions that induce activation. However, due to the lack of strictly defined criteria for macrophage phenotyping, there is now a partial consensus on the phenotyping of macrophages. According to their different activation states, macrophages are mainly classified as either M1‐type macrophages (also known as classically activated macrophages with antitumor activity) or M2‐type macrophages (also known as alternatively activated macrophages with protumor activity).^[^ ^27^ ^]^ Currently, the simplest and clearest way to distinguish the polarization state of macrophages is based on the differences in gene and protein expression between phenotypes. The differences in the gene expression profiles of M1‐polarized and M2‐polarized macrophages have been refined over time^9^. In this study, we detected the M2‐TAM markers CD163, IL‐10, IL1RN, TGFB1, and CCL2 and the M1‐TAM marker TNFA by qRT‒PCR (Figure S1a, Supporting Information).

In chronic inflammation, such as that caused by cancer, autoimmune disease, or infection, the inflammatory site will continuously secrete inflammatory factors and other substances to stimulate the bone marrow to continuously export monocytes, which localize to the inflammatory site through chemotaxis and maintain the inflammatory state at the site.^[^ ^28^ ^]^ For example, tumors maintain large intratumoral macrophage populations through the continuous recruitment of monocytes in the blood.^[^ ^29^ ^]^ What are the characteristics of macrophage polarization in patients with chronic inflammation? This question is difficult to answer systematically. The process of chronic inflammation spans months, years, and even decades, resulting in macrophages that continue to receive inflammatory activation signals over long periods of time. In the case of tumors, monocytes accumulate in the tumor bed through the CCL2‐CCR2 signaling axis.^[^ ^30^ ^]^ Tumors undergo a process of growth, hypoxia, and remodeling during development. This process is accompanied by changes in the tumor microenvironment, and monocytes entering the tumor are a part of this change as they transition from ly6C+ cells to mature macrophages; the macrophages can even die and be replaced by new monocytes that are dynamically developed.^[^ ^29^, ^30^ ^]^ Due to the complexity of the tumor microenvironment, the polarization of macrophages in tumors is more complex, often with mixed features of the M1‐ and M2‐type macrophages.^[^ ^30^, ^31^ ^]^ Thus, in cases of chronic inflammation, macrophage polarization can take months, years, or even longer. Because of this long length of time, it is difficult to connect the whole process of macrophage polarization linearly. Recently, a growing body of research has shown that exosomes may play a key role in orchestrating the crosstalk between macrophages and cancer cells. As an extracellular carrier, exosomes transport a variety of lipid molecules (including lipids, proteins, nucleic acids, etc.) to target cells for different roles. Macrophage‐derived exosomes are heterogeneous in different cancers and they play paradoxical roles in suppressing and promoting tumors primarily through posttranscriptional control and regulation of protein phosphorylation in recipient cells. Moreover, exosomes secreted by macrophages with different phenotypes have a variety of therapeutic impacts. Therefore, understanding the secretion of different types of macrophages and their corresponding exosomes in cancer is potentially applicable in the clinic.^[^ ^32^ ^]^ Herein, we demonstrated that M2 macrophage‐derived exosomes carrying LINC01232 promote tumor immune escape and knockdown of LINC01232 inhibited tumor immune escape. After LINC01232 knockdown, we administered activated CD8^+^ T cells by tail vein infusion. Compared with the control group, the LINC01232 knockdown group was more sensitive to CD8^+^ T cell treatment. Importantly, knockdown of LINC01232 enhanced MHC‐I expression on the tumor cell surface, whereas overexpression of LINC01232 attenuated MHC‐I expression (Figure S10, Supporting Information).

TAMs can promote tumorigenesis and cancer progression through immune and nonimmune processes. Studies have reported that TAMs promote tumor angiogenesis and blood‐derived cell metastasis by secreting a large amount of proangiogenic factors, such as vascular endothelial growth factor (VEGF).^[^ ^33^ ^]^ TAMs can also produce immunosuppressive factors, such as IL‐10, TGF*β*, and PGE2, etc., which promote the occurrence and development of tumors by inhibiting the antitumor immune response. Among them, IL‐10 significantly reduces the effect of antitumor therapy by inhibiting the antitumor immune response mediated by chemotherapeutic drugs.^[^ ^34^ ^]^ TAMs in glioblastoma tumor tissues secrete a large amount of pleiotrophin (PTN) protein, while glioblastoma cancer stem cells (CSCs) contain a large amount of its receptor PTPRZ1. The combination of the two can activate a series of signaling pathways, generate CSCs, and maintain their malignant behavior, promote tumor growth and progression, and lead to increased mortality in patients. This finding reveals the role of the special immune microenvironment that is formed by paracrine TAMs to promote the malignant behavior of CSC‐initiated gliomas.^[^ ^35^ ^]^ An increasing number of studies have found that TAMs can deliver miRNAs to tumor cells by secreting exosomes, thereby inducing drug resistance in tumor cells. TAM‐derived exosomes significantly reduce the sensitivity of pancreatic ductal adenocarcinoma to gemcitabine in vivo and in vitro by transferring miR‐365.^[^ ^36^ ^]^ Therefore, targeting exosomal miRNAs may be a strategy for tumor diagnosis and treatment.^[^ ^37^ ^]^ Herein, we demonstrated that targeting the M2 macrophage‐derived exosome LINC01232 inhibits tumor immune escape.

In addition to targeting exosomes, immunotherapy targeting various molecular switches and proteins has also been effective. PI3K*γ* is a molecular switch that can eliminate the immunosuppressive function of TAMs by turning off TAM immunomodulatory activity. Studies have found that macrophages with PI3K*γ* inactivation highly express MHC class II molecules and proinflammatory cytokines such as interleukin 12 (IL‐12), while they express low levels of the immunosuppressive molecules IL‐10, and Arginase, etc. In a series of tumor models, inhibition of the PI3K*γ*‐mediated activity of TAMs can activate adaptive immunity, enhance the recruitment, and activity of cytotoxic T lymphocytes, and thus significantly inhibit cancer cell growth and metastasis. These results show that PI3K*γ* inhibitors targeting TAMs may significantly enhance the efficacy of immunotherapy.^[^ ^38^ ^]^ Targeting macrophage‐derived granulin can be a strategy to improve or restore T‐cell infiltration and cytotoxic function in pancreatic cancer, and as such, this is a potential antitumor therapeutic strategy.^[^ ^39^ ^]^ Researchers have designed the inhibitor AK750, which not only prevents macrophages from receiving macrophage colony‐stimulating factor (MSF) from cancer cells, but also prevents cancer cells from releasing CD47 protein. This makes it difficult for cancer cells to send the “do not eat me” message to macrophages. In mice injected with AK750, tumor cell growth was significantly inhibited in both melanoma and breast cancer.^[^ ^40^ ^]^ Thus, the tumor‐promoting functions of TAMs are critical for the growth and metastasis of most solid tumors. Understanding how to inhibit the tumor‐promoting function of TAMs and turn them from tumor‐promoting to antitumor cells will be important for future tumor immunotherapy strategies.

Tumor immunotherapy is a treatment method to control and eliminate tumors by restarting and maintaining the tumor‐immune cycle and restoring the body's normal antitumor immune response. Immunotherapy has proven effective in many malignancies.^[^ ^41^ ^]^ Tumor immunotherapy works synergistically with a variety of important proteins to improve or restore the function of immune cells in the tumor microenvironment and it has become a major driving force for personalized medicine.^[^ ^42^ ^]^ In recent years, with the in‐depth study of tumor immune escape mechanisms, new ideas for the study of tumor immunotherapy have been reported. For example, some researchers recently found that there is a correlation between EMT and PD‐L1 expression, and tumor cells with epithelial properties express less PD‐L1. After EMT transformation, tumor cells with mesenchymal properties have increased PD‐L1 expression and are more likely to escape the surveillance of the immune system.^[^ ^43^ ^]^ Most cancer immunotherapies, including immune checkpoint blockade therapy, aim to counter immune evasion by shifting the balance in favor of immune activation, allowing T‐cell‐mediated elimination of cancer cells.^[^ ^44^ ^]^ However, only a small fraction of patients benefit from immunotherapy, so there is an urgent need to identify the genomic and molecular determinants that are used to support immune evasion.^[^ ^19a^ ^]^ FS118 is a novel bispecific antibody that adds a LAG‐3‐binding Fc region to a PD‐L1‐specific IgG1 antibody, potentially providing excellent antitumor effects while suppressing adverse reactions through dual targeting. An FIH phase I study with this agent (NCT03440437) is ongoing in adult patients with solid tumors for whom prior PD‐1/PD‐L1 therapy has failed.^[^ ^45^ ^]^ T‐cell immunoglobulin and mucin domain‐3 (Tim‐3) is a cell surface protein that is involved in T‐cell exhaustion and resistance to ICIs. Monoclonal antibodies against TIM3 reduced Treg activation and decreased CTLA4 and TIGIT expression. A phase I clinical trial of the anti‐TIM3 antibody TSR‐022 is underway (NCT02817633). Preliminary phase Ia/Ib results of LY3321367 showed that the drug was well tolerated as a monotherapy and in combination with anti‐PD‐L1 LY3300054 (anti‐PD‐L1).^[^ ^46^ ^]^ In this study, we found that LINC01232 derived from M2‐TAM exosomes promoted tumor immune escape through the E2F2/NBR1/MHC‐I signaling axis. Disruption of E2F2/NBR1/MHC‐I signaling by shRNA or blockade of the corresponding antibodies largely abolished the tumor‐supportive effects of LINC01232 and inhibited tumor growth driven by M2‐type macrophages. Importantly, knockdown of LINC01232 enhanced the expression of MHC‐I on the surface of tumor cells and improved the response to reinfusion with CD8^+^ T cells (Figure S11, Supporting Information).

## 4 Experimental Section

### Clinical Samples

Glioma surgical specimens were collected from Wuhan Union Hospital (Wuhan, China). Tables S1 and S2 (Supporting Information) provide details on patient characteristics. Before specimen collection, all patients signed informed consent forms. This study was conducted in accordance with the guidelines in the Declaration of Helsinki, and the relevant ethical approval was obtained. Please refer to the Supporting Information for further details.

### Cell Culture and Treatment

The method described in the previous publications ^[^ ^26^, ^47^ ^]^ was used for cell culture. THP1 cells were induced to differentiate into M0‐TAMs by treatment with 100 ng mL^−1^ PMA and were induced to differentiate into M2‐TAMs by simultaneous treatment with 100 ng mL^−1^ phorbol ester (PMA) and 20 ng mL^−1^ IL‐4. Short tandem repeat analysis was run on all cells and they were regularly tested for mycoplasma contamination. Detailed methods are provided in the Supporting Information.

### Plasmids, Small Interfering RNAs (siRNAs), and Transfection

GeneChem Co. Ltd. (Shanghai, China) provided all shRNAs (Table S3, Supporting Information). All protocols were performed according to the manufacturer's instructions. The Supporting Information provides detailed methods.

### Western Blotting (WB)

The related protocol was published in detail in the previous studies.^[^ ^26^, ^47^ ^]^ The details of all antibodies used were listed in Table S4 (Supporting Information).

### Real‐Time Quantitative RT‐PCR (qRT‐PCR)

This assay was performed according to the manufacturer's instructions. The 2^−ΔΔCt^ method was used to normalize the expression data to that of GAPDH, which served as the control.^[^ ^48^ ^]^ GeneCreate (Wuhan, China) was used to synthesize the primers for this study. Primer sequences are listed in Table S5 (Supporting Information) and the Supporting Information provide more detailed methods.

### Bioinformatic Analysis

TCGA (https://cancergenome.nih.gov/) and Genotype Tissue Expression (GTEx) databases were used to acquire data.^[^ ^49^ ^]^ The results from independent sample t tests comparing two groups were presented. The Supporting Information provides a detailed description of the methodology.

### Isolation and Identification of Exosomes

Standard operating procedures were followed for ultracentrifugation to purify the exosomes. Please refer to the previous articles for more detailed protocols.^[^ ^26^, ^47^ ^]^

### Chromatin Immunoprecipitation (ChIP)

ChIP assay kits (Upstate Biotechnology, Temacula, CA) were used. Following formaldehyde crosslinking, the cells were sonicated. Incubation with anti‐E2F2 antibodies was performed after pretreatment with protein A/G beads. As a negative control, IgG was used. In this study, DNA was extracted from complexes using DNA extraction kits (QIAGEN), and quantitative real‐time PCR was performed. A list of the primers used in ChIP‒qPCR can be found in Table S6 (Supporting Information).

### Dual Luciferase Reporter Assays

A dual‐luciferase reporter assay was performed in accordance with the manufacturer's instructions (Promega). In brief, luciferase reporters were constructed by annealing complementary oligonucleotides containing putative NBR1 binding sites (wild type (wt) and mutant type (mut)) and inserting them into the pGL3‐control firefly luciferase reporter gene vector. Please refer to the previous article for more details regarding the protocol.^[^ ^26^, ^47^ ^]^

### Subcellular Fractionation Analysis

The PARISTM Kit (Ambion, Austin, TX) was used for the subcellular fractionation of RNAs. In addition, qRT‒PCR was used to determine the RNA content in the cytoplasm and nucleus. *β*‐actin and U6 served as internal references for the cytoplasm and nucleus, respectively. By using the Minute Cytoplasmic and Nuclear Extraction kit (Invent Biotechnologies), proteins were separated and extracted from the cytoplasm and nucleus. *α*‐Tubulin and lamin B1 were the internal references for the cytoplasm and nucleus, respectively.

### Fluorescence in situ Hybridization (FISH), Immunofluorescence (IF), and Immunohistochemistry (IHC)

In tissue sections, LINC01232 detection probes (RIBOBIO, Guangzhou, China) and FISH kits (Bosterbio, USA) were used to perform FISH assays. FISH, IF, and IHC assays and scoring techniques were conducted as previously described.^[^ ^26^ ^]^ Table S4 (Supporting Information ) lists the antibodies used in this experiment.

### Co‐IP (Coimmunoprecipitation)

Co‐IP assays were conducted as reported previously.^[^ ^26^, ^47^ ^]^ Detailed information about the antibodies used in this experiment can be found in Table S4 (Supporting Information).

### Proximity Ligation Assay (PLA)

A Duolink In Situ Red Starter Kit Mouse/Rabbit (Sigma‒Aldrich, St Louis, MO, USA) was used for PLA. Briefly, 4% paraformaldehyde was used to fix U‐87MG cells on glass coverslips. After being permeabilized with 0.1% Triton X‐100, the cells were blocked for 1 h with blocking solution. The cells were then incubated overnight at 4 °C with antibodies against E2F2 and NBR1 (Abcam, Cambridge, USA). Then, the PLA probes were added to the primary antibodies to bind with the corresponding positive or negative strands. At room temperature, the cells were sequentially incubated with ligase for 0.5 h and polymerase for 2 h. Duolink in situ Mounting Medium with DAPI was then used to mount the coverslips on the slides. In the previous article,^[^ ^26^ ^]^ the protocol was discussed in greater detail.

### Biotin‐RNA Pull‐Down Assays

In brief, full‐length LIN01232 sequences were amplified using PCR and then reverse transcribed. Proteins from cells were lysed with lysis buffer. Next, streptavidin agarose beads were used to capture biotin‐labeled LIN01232 probes from the samples. Mass spectrometry or WB was used to measure the pull‐down complexes. The protocol was described in more detail in the previous article.^[^ ^26^ ^]^

### RIP Assays

RIP assays were conducted using the Magna RNA‐binding protein immunoprecipitation kit (Millipore, MA, USA). Cells were collected and lysed using RIPA buffer. The cell lysates were then incubated with RIP buffers containing magnetic beads conjugated to human anti‐E2F2 or IgG antibodies. Then, the levels of coprecipitated RNAs were measured by qRT‒PCR. To confirm that the detected RNA signal was linked to E2F2, total RNA and IgG controls were also measured. The previous article provides more details regarding the protocol.^[^ ^26^ ^]^

### Brain Orthotropic Xenografts

In this study, BALB/c nude mice (6–8 weeks old) were used. U‐87MG and U‐251 cells were injected stereotactically into the brain after resuspending them in cold PBS. D‐luciferin (15 mg mL^−1^, 200 mL) was then injected intraperitoneally and used IVIS to image live animals. Animal experiments were conducted as described in the previous papers.^[^ ^26^, ^47^ ^]^

### Statistical Analysis

R 4.0.2 software (http://www.r‐project.org/) and GraphPad Prism (version 8.0; GraphPad Inc., La Jolla, CA, USA) were used to perform all statistical analyses. More details can be found in the Supporting Information.

### Ethical Approval and Consent to Participate

The Ethics Committee of Wuhan Union Hospital (S0608) approved all aspects of the study, and informed consent was obtained from all patients. The Tongji Medical College's Institutional Animal Care and Research Advisory Committee approved all animal experiments conducted in the laboratory (S2838).

## Conflict of Interest

The authors declare no conflict of interest.

## Author Contributions

J‐J.L., X.W., and X‐B.J. designed and performed the experiments. X‐B.J., X.W., and J.R. provided clinical samples and patient information. N.T, Z‐J.Z., C.J., M‐J.W., and K.Z. analyzed the data. K‐S.W., C.Y., S‐Q.W., P‐W.S., and H‐L.Y. provided advice and provided technical assistance. J‐J.L. and X.W. wrote the manuscript. J‐J.L. and J.R. completed the drawing. J‐J.L., J‐S.L., Z‐Y.X., T.L., and X‐B.J. critically reviewed the manuscript.

## Supporting information

Supporting Information

## Data Availability

The data that support the findings of this study are available from the corresponding author upon reasonable request.
