# Correction to [Tumor‐Associated Macrophage‐Derived Exosomes LINC01232 Induce the Immune Escape of Glioma by Decreasing MHC‐I Surface Expression]

**DOI:** 10.1002/advs.202500268

**Published:** 2025-01-28

https://doi.org/10.1002/advs.202207067

In the original version of our article, there were errors in Figures 2, 4, 5, S8, S9, S10, S12, and S13. Specifically, the representative image of β‐actin cells in Figure 2F, the representative image of GAPDH groups in Figure 4B–D, the representative image of si‐LINC01232#1 groups in Figure 5D, the representative image of lamin B1 groups in Figure S8A, the representative image of 1232‐OE/U‐87MG groups in Figure S9B, the representative image of GAPDH/U‐251 groups in Figure S10B, the representative image of M0‐Exos/T98G groups in Figure S12F and the representative image of LINC01232‐OE+NBR1‐KD groups in S13D are incorrect. The correct images are provided below. This correction will not affect the results and conclusions. The authors apologize for any inconvenience this may have caused.

The representative image of β‐actin cells in Figure 2F was incorrect.

“
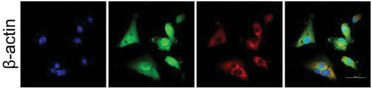
”

This should have read:

“
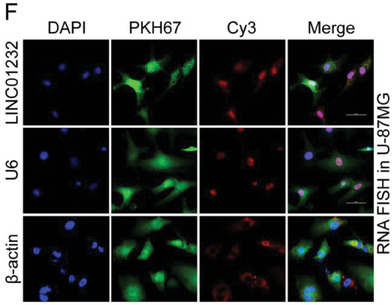
”

The representative image of GAPDH groups in Figure 4B–D was incorrect.

“
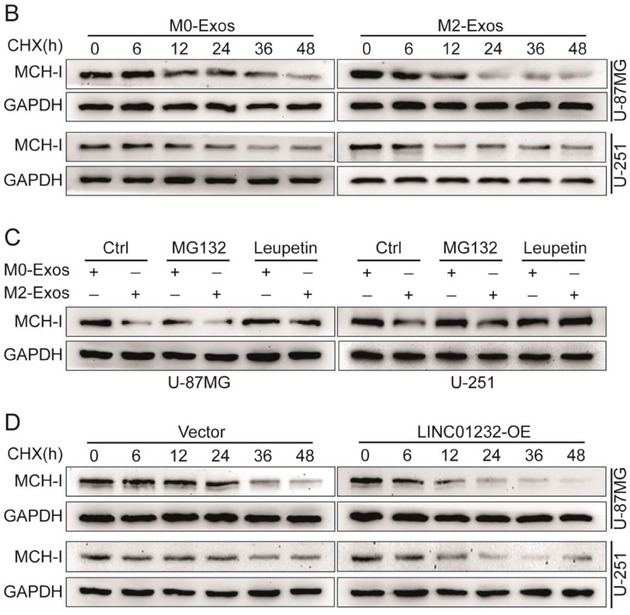
”

This should have read:

“
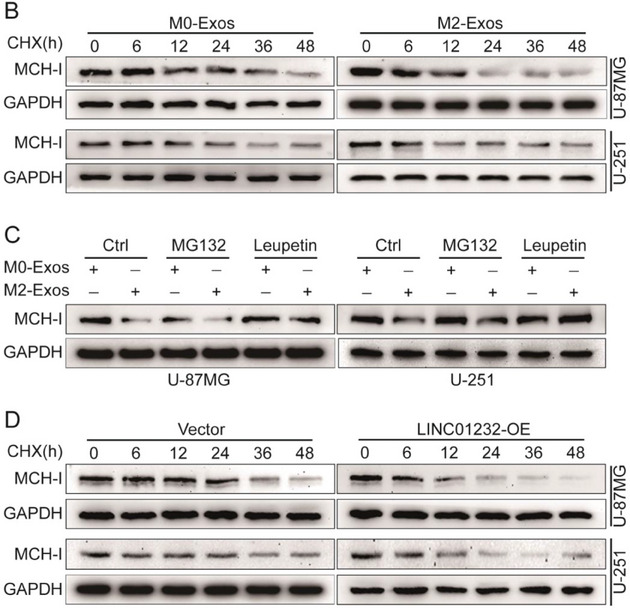
”

The representative image of si‐LINC01232#1 groups in Figure 5D was incorrect.

“
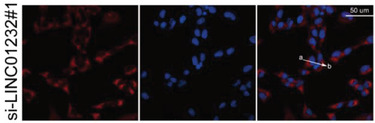
”

This should have read:

“
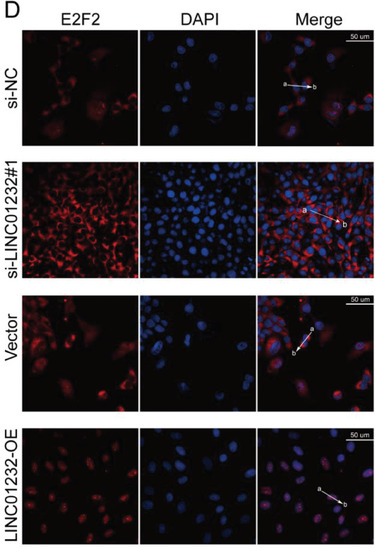
”

The representative image of lamin B1 groups in Figure S8A was incorrect.

“
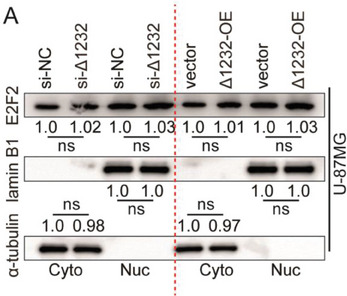
”

This should have read:

“
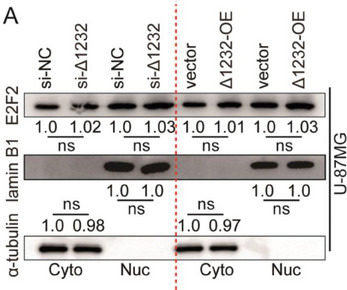
”

The representative image of 1232‐OE/U‐87MG groups in Figure S9B was incorrect.

“
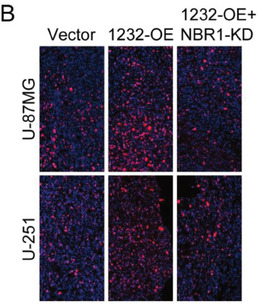
”

This should have read:

“
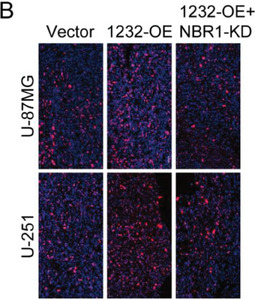
”

The representative image of GAPDH/U‐251 groups in Figure S10B was incorrect.

“
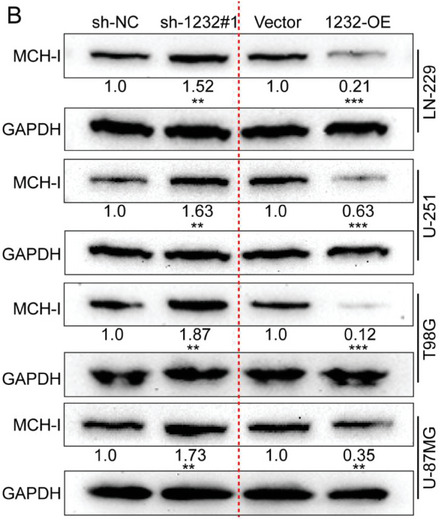
”

This should have read:

“
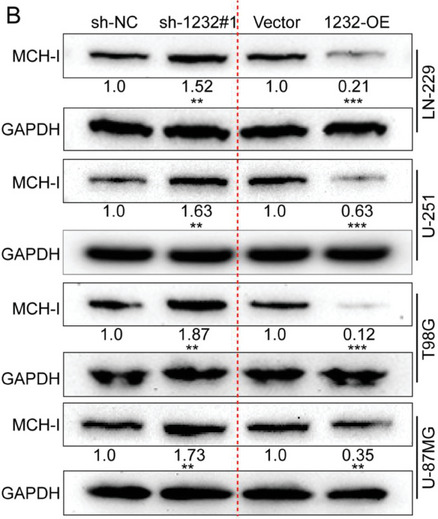
”

The representative image of M0‐Exos/T98G groups in Figure S12F was incorrect.

“
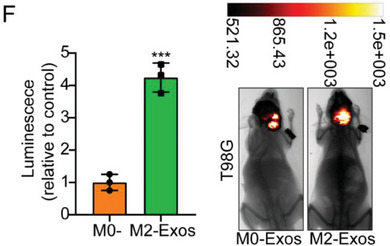
”

This should have read:

“
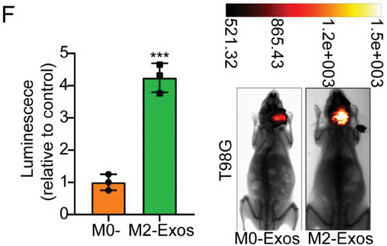
”

The representative image of LINC01232‐OE+NBR1‐KD groups in Figure S13D was incorrect.

“
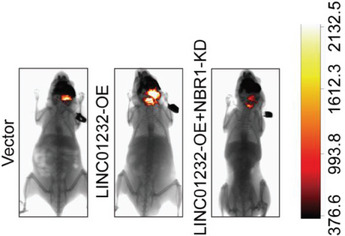
”

This should have read:

“
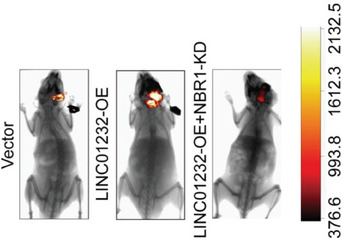
”

The correct image is shown below


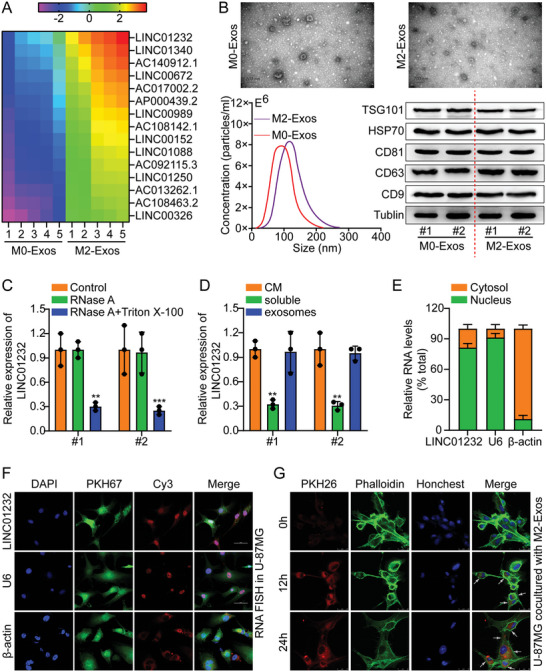


Correct image of Figure 2


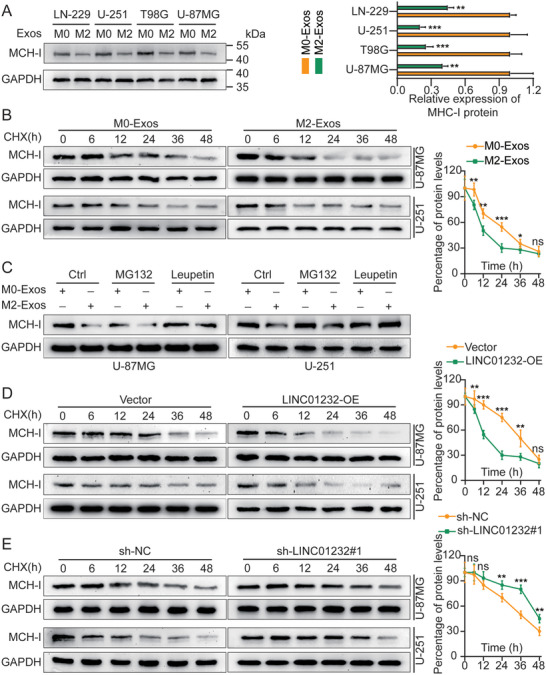


Correct image of Figure 4


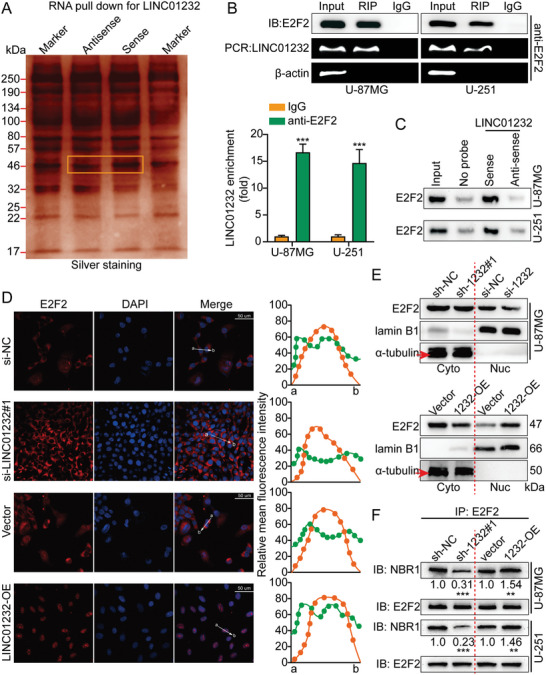


Correct image of Figure 5


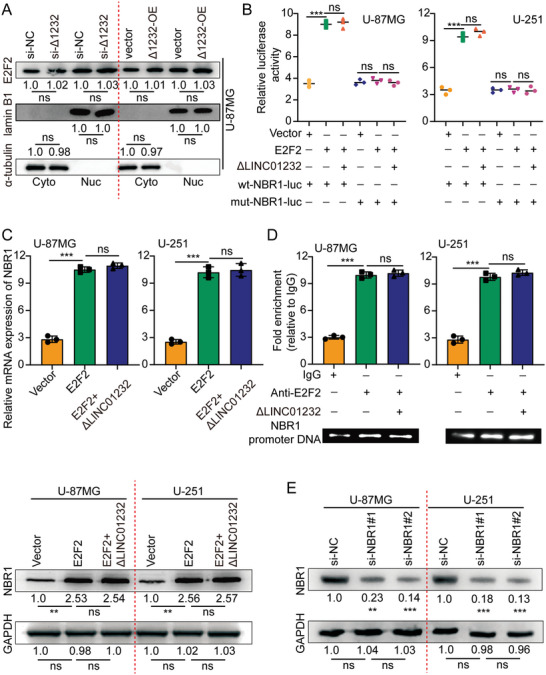


Correct image of Figure S8


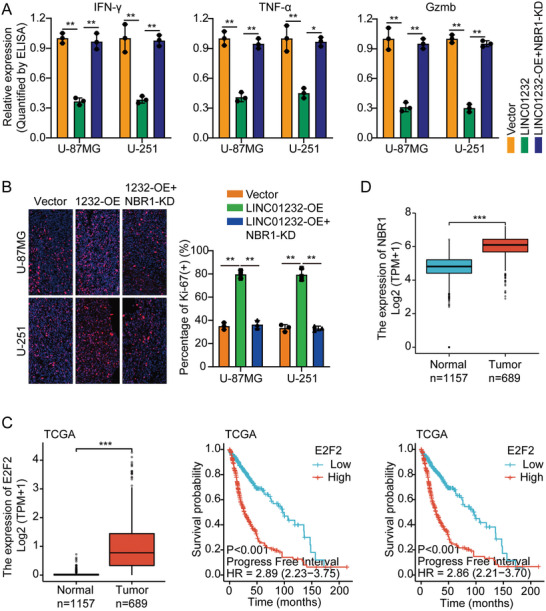


Correct image of Figure S9


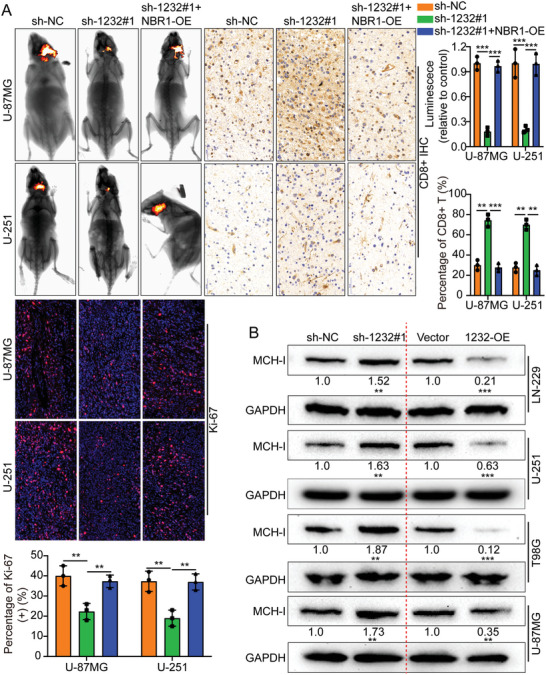


Correct image of Figure S10


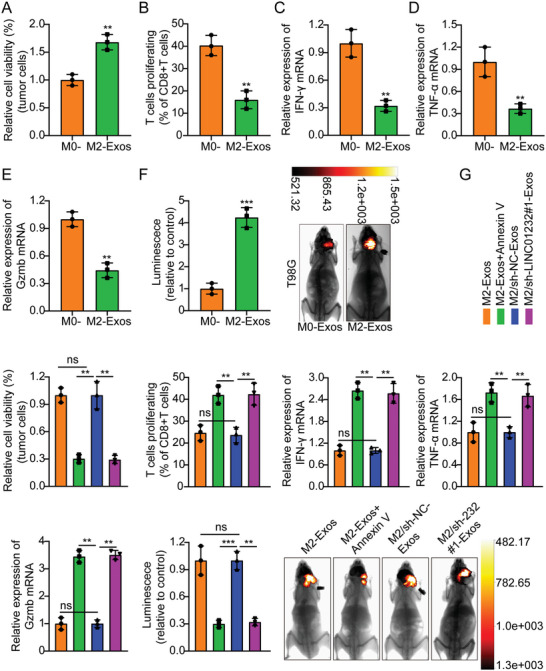


Correct image of Figure S12


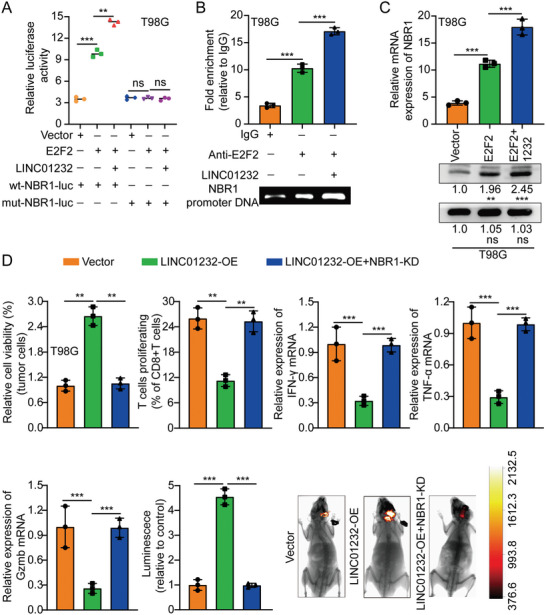


Correct image of Figure S13

