# Supplementary material for: Tumor‐Associated Macrophage‐Derived Exosomal LINC01232 Induces the Immune Escape in Glioma by Decreasing Surface MHC‐I Expression
Source: Adv Sci (Weinh). 2023 Apr 25;10(17):2207067. doi: 10.1002/advs.202207067 (PMC10265094; doi:10.1002/advs.202207067)
Supplement: Supplementary file 1 — Supporting Information [file ADVS-10-2207067-s001.pdf]

ADVANCED SCIENCE
Open Access

Supporting Information

for *Adv. Sci.*, DOI 10.1002/advs.202207067

Tumor-Associated Macrophage-Derived Exosomal LINC01232 Induces the Immune Escape in Glioma by Decreasing Surface MHC-I Expression

Junjun Li, Keshan Wang, Chao Yang, Kai Zhu, Cheng Jiang, Minjie Wang, Zijie Zhou, Nan Tang, Qiangping Wang, Siqi Wang, Pengwei Shu, Hongliang Yuan, Zhiyong Xiong, Jinsong Li, Tao Liang, Jin Rao, Xuan Wang and Xiaobing Jiang**

Tumor-associated macrophage-derived exosomal LINC01232 induces immune escape in glioma by decreasing surface MHC-I surface expression

Junjun Li[1], Keshan Wang[2], Chao Yang[3], Kai Zhu[1], Cheng Jiang[1], Minjie Wang[1], Zijie Zhou[1], Nan Tang[1], Qiangping Wang[1], Siqi Wang[4], Pengwei Shu[4], Hongliang Yuan[5], Zhiyong Xiong[1], Jinsong Li[6], Tao Liang[7], Jin Rao[1], Xuan Wang[1#], Xiaobing Jiang[1#]

Methods

Patients and clinical samples

All fresh glioma specimens were obtained from the patients operated at the Wuhan Union Hospital. All specimens were in quadruplicates, each stored in (i) tissue preservation solution for primary culture, (ii) 4% paraformaldehyde for fixation, (iii) in situ hybridization preservation solution for fixation, and (iv) liquid nitrogen for subsequent experiments. Before collecting the specimens, we obtained "informed written consent" from patients. No patient received radiotherapy or chemotherapy before the surgery. This research was performed according to the International Ethical Guidelines for Biomedical Research Involving Human Subjects issued by the Council for International Organization of Medical Sciences (CIOMS). Details on patient characteristics are provided in Supplementary Tables 1-2. Before specimen collection, all patients signed informed consent. The study was conducted in accordance with the guidelines in the Declaration of Helsinki and the relevant ethical approvals were obtained. All patients were graded according to the 2016 edition of the World Health Organization (WHO) classification criteria for central nervous system (CNS) tumor.

Cell culture and treatment

Normal human astrocyte (NHA) and human astrocyte (HA) cells were obtained from the ScienCell Research Laboratories (San Diego, CA) and cultured in the astrocyte medium (Carlsbad, CA, USA). Human glioma cell lines (LN-229, T98G, H4, LN-18, A-172, U-118MG, U-251MG, and U-87MG (glioblastoma of unknown origin)) were obtained from the American type culture collection (ATCC). All cell lines were authenticated by short tandem repeat (STR) and verified to be free of mycoplasma contamination. THP1 cells were induced to differentiate into M0 TAMs by treatment

with 100ng/mL PMA, and were induced to differentiate into M2 TAMs by simultaneous treatment with 100ng/mL phorbol ester (PMA) and 20ng/mL IL-4. For detailed cell culture protocol, please refer to our previous articles [1].

Plasmids, small interfering RNA (siRNA), and transfection

The U-87MG and U-251 cells were transfected with the shRNAs targeting LINC01232, E2F2 and NBR1 using Lipofectamine 3000 (Invitrogen). We constructed the LINC01232 pcDNA3.1 (1232-OE (overexpression)), E2F2 pcDNA3.1 (E2F2-OE), and NBR1 pcDNA3.1 (NBR1-OE) inserting the respective genes into the pcDNA3.1 vector (Invitrogen). Empty pcDNA3.1 vector (Vector) was used as control. All the siRNAs and sh1232 and shNC lentiviral vectors were obtained from GeneChem Co., Ltd (Shanghai, China). Supplementary Table 3 lists the sequences of relative siRNAs and shRNAs. For detailed protocol, please refer to our previous articles [1a].

Real-time quantitative RT-PCR (qRT-PCR)

Total RNA was isolated from the cells and exosomes using TRIzol and TRIzol LS reagents (Life Technologies). The miRNAs were reverse transcribed using the Mir-X™ miRNA First-Strand Synthesis Kit (Clontech, Mountain View, CA, USA). Real-time PCR was performed using the SYBR Green PCR Master Mix (Takara, Shiga, Japan) and the primers listed in Supplementary Table 5. The mRNA levels were measured with the 7500 Fast Real-Time PCR Systems (Applied Biosystems, Foster City, CA, USA). GAPDH was used as internal control.

Bioinformatic analysis

All datasets were downloaded from The Cancer Genome Atlas (TCGA) **(**https://cancergenome.nih.gov/**)** and Genotype Tissue Expression (GTEx) database [2]. UCSC XENA (https://xenabrowser.net/datapages/) RNAseq data in TPM format of TCGA and GTEx uniformly processed by the Toil process [3]. RNAseq data in TPM (transcripts per million reads) format and log2 transformation for expression comparison between samples. RNAseq data in FPKM (Fregments Per Kilobase per Million) format was converted into TPM (transcripts per million reads) format and log2 conversion was performed. Prognostic data from the following related articles [4]. The results of the independent sample *t*-tests comparing the two groups are

expressed as *t*-test values.

Statistical analysis

All statistical analyses were conducted with SPSS 23.0 software (SPSS, Inc., Chicago, IL), R 4.0.2 software (http://www.r-project.org/) and GraphPad Prism (version 8.0; GraphPad Inc., La Jolla, CA, USA). All data were presented as the mean ± sd. Unpaired/paired Student's t test for two groups or one-way ANOVA + Dunnett's for more than two groups, which was used to assess statistically significant data. The chi-square test, Pearson's correlation, and one-way analysis of variance were also performed. Cox regression analysis and Log-rank test were used to determine survival difference and hazard ratio. P-values < 0.05 were considered to be statistically significant.

Supplementary Tables 1: Correlation of the expression levels of LINC01232 in glioma tissues with clinicopathologic features.

**Characteristics**	**No. of cases (%)**	**LINC01232**		**P-value**
		**Low**	**High**	
**Age (y)**				
＜50	30 (37.5%)	10	20	0.149
⩾50	50 (62.5%)	25	25	
**Gender**				
Male	35 (43.8%)	15	20	0.892
Female	45 (56.2%)	20	25	
**Tumor size (cm)**				
＜2	48 (60.0%)	28	20	0.002**
⩾2	32 (40.0%)	7	25	
**Tumor location**				
Supratentorial	42 (52.5%)	18	24	0.857
Subtentorial	38 (47.5%)	17	21	
**Karnofsky performance scale**				

＜90	46 (57.5%)	13	33	0.001**
⩾90	34 (42.5%)	22	12	
**WHO grade**				
I	8 (10.0%)	6	2	0.033*
II	27 (33.8%)	10	17	
III	20 (25.0%)	12	8	
IV	25 (31.2%)	7	18	
**Tumor recurrence**				
No	40 (60.0%)	23	17	0.011*
Yes	38[#] (40.0%)	11[#]	27[#]	

Partial data not available; statistics based on available data. *<0.05 and **<0.01.

LINC01232 high expression: score 8-16; low expression: score 0-7.

Supplementary Tables 2: Univariate and multivariate for clinicopathological features associated with various prognostic parameters of 80 glioma patients by Cox-regression analysis.

Variables	Univariate analysis		Multivariate analysis	
	HR (95%CI)	P-value	HR (95%CI)	P-value
Age (⩾50 vs ＜50)	1.426 (1.037-2.528)	0.427	1.754 (1.273-2.962)	0.638
Gender (Male vs Female)	1.582 (1.165-3.264)	0.763	1.735 (1.291-3.375)	0.859
Tumor size (⩾2 vs ＜2)	2.353 (1.258-4.273)	0.008**	1.942 (1.493-3.762)	0.015*
Tumor location (Sup vs Sub)	1.652 (1.257-2.796)	0.582	1.725 (1.386-3.215)	0.816
KPS (⩾90 vs ＜90)	1.972 (1.241-3.693)	0.012**	1.572 (1.031-2.851)	0.026*
WHO grade (HGG vs	2.341	0.038*	2.158	0.042*

LGG)	(1.592-3.846)		(1.425-4.315)	
Tumor recurrence (Yes vs No)	1.032 (0.642-2.431)	0.025*	1.217 (0.865-2.869)	0.032*
LINC01232 (Low vs High)	2.573 (1.576-4.586)	0.005**	2.264 (1.352-3.968)	0.014*

Supplementary Tables 3: Gene primer series

**Gene**	**Primer**	**Sequence(5′-3′)**
si-1232#1	forward	CACTTCATTTACATGGGTGCG
	reverse	ACCCGTAGAGGGTCTTGAGTG
si-1232#2	forward	GCATAGGCGTGTGACCTTT
	reverse	GCGTGTGACCTTTGTAACT
si-E2F2#1	forward	CGTCCCTGAGTTCCCAACC
	reverse	GCGAAGTGTCATACCGAGTCTT
si-E2F2#2	forward	TCCATGAACAGATCGTCATTGC
	reverse	TCCGTTGGTGCTCCTTATGTG
si-NBR1#1	forward	AGATGGCAGTTAAACAGGGAAAC
	reverse	GTGGGGCTTCATCAACGACA
si-NBR1#2	forward	CAGTGCAGTCGTTTCCACTTG
	reverse	AGGTAGCTTGTGAACCAGTCT
sh-1232	GGACTCTAATACGACTCACTATAGGG	
	AGAAGTGCAACTTTGTAACTTCAC	

Supplementary Tables 4: Protein Antibody Information

Name	Description
Anti-E2F2 antibody	ab138515 (Abcam, Cambridge, USA)
Anti-NBR1 antibody	ab55474 (Abcam, Cambridge, USA)
Anti-MHC-I antibody	Cat. No. sc-32235 (Santa Cruz, Texas, USA)

Anti-CD9 antibody	ab236630 (Abcam, Cambridge, USA)
Anti-CD63 antibody	ab134045 (Abcam, Cambridge, USA)
Anti-CD81 antibody	ab79559 (Abcam, Cambridge, USA)
Anti-HSP70 antibody	ab2787 (Abcam, Cambridge, USA)
Anti-TSG101 antibody	ab125011 (Abcam, Cambridge, USA)
Anti-GAPDH antibody	#5174 (Cell Signaling Technology, Beverly, MA, USA)
Anti-Calnexin antibody	10427-2-AP (Proteintech, Wuhan, China)
Anti-Alpha Tubulin antibody	66031-1-lg (Proteintech, Wuhan, China)
Anti-Lamin B1 antibody	#13435 (Cell Signaling Technology, Beverly, MA, USA)

Sequences of probes for LINC01232 RNA FISH

5’-DIG-GGACTCTAATACGACTCACTATAGGGAGAAGTGCAACTTTGTAACTTCAC-DIG-3’

Supplementary Tables 5: Gene primer series

**Gene**	**Primer**	**Sequence(5′-3′)**
LINC01232	forward	CACTTCATTTACATGGGTGCG
	reverse	ACCCGTAGAGGGTCTTGAGTG
E2F2	forward	TCCATGAACAGATCGTCATTGC
	reverse	TCCGTTGGTGCTCCTTATGTG
NBR1	forward	AGGAGCAAAACGACTAGCTGC
	reverse	TCTGGGGTCTTCATGTCTGAT
MHC-I	forward	CCATGAGGTATTTGTGGACCG
	reverse	TCTCGGACTCTCGTCGTCG

β-actin	forward	ATTGCCGACAGGATGCAGAA
	reverse	GCTGATCCACATCTGCTGGAA
GAPDH	forward	GAGTCAACGGATTTGGTCGT
	reverse	TTGATTTTGGAGGGATCTCG

Supplementary Tables 6: Gene primer series

Gene	Primer	Sequence(5′-3′)
ChIP-NBR1 promoter 1-1	forward	GCGGCTTATTACGTCACATTAATTGCTGTACC
	reverse	CGCCGAATAATGCAGTGTAATTAACGACATGG
ChIP-NBR1 promoter 1-1	forward	GAGTGTTGTTATGTTCTCCTATCTTGAGAGCA
	reverse	CTCACAACAATACAAGAGGATAGAACTCTCGT
ChIP-NBR1 promoter 2-1	forward	ACCTACGGCTGCGAAACACAATTACAATATCC
	reverse	TGGATGCCGACGCTTTGTGTTAATGTTATAGG
ChIP-NBR1 promoter 2-2	forward	TAGAATTATTCTCATAATTCTTATTCTCAT
	reverse	ATCTTAATAAGAGTATTAAGAATAAGAGTA

Supplementary Tables 7: The top 10 upregulated lncRNA in RNA-seq.

ensembl_gene_id	Gene_Name	Gene_Type	log2FC	Fold_Change	p_value	q_value
ENSG00000250331	LINC01340	lincRNA	7.41130283304775	1.86414233803103	0.00346460293478399	0.0486586773264469
ENSG00000271943	AC140912.1	lincRNA	7.43366174632833	8.8978499649914	0.00338973632913729	0.0478835390573655
ENSG00000280734	LINC01232	lincRNA	6.97750259797837	2.29298501696153	0.00336814798106576	0.0476972310934633
ENSG00000263874	LINC00672	lincRNA	6.8646279263917	3.82567489637925	0.00150808246276135	0.0261094439386204

ENSG00000250334	LINC00989	lincRNA	5.27638433457581	2.952211851771992	0.00203764086509183	0.0326559264136448
ENSG00000215386	AC017002.2	lincRNA	1.41286631742744	1.922102373772272	0.000150743916534255	0.00421852364795352
ENSG00000258850	AP000439.2	lincRNA	7.70977862588978	5.00873654431616	0.00115691726469176	0.0215401557609714
ENSG00000229603	AC108142.1	lincRNA	4.1973077253825	1.69855829490564	0.00140541501363001	0.0247341427398769
ENSG00000240996	LINC00152	lincRNA	7.57921581559251	5.15500094125212	0.00171260084359624	0.0289443134637953
ENSG00000249307	LINC01088	lincRNA	3.19647843322519	1.194001824343452	0.0030840481944413	0.0446387808330518

Supplementary figure legends

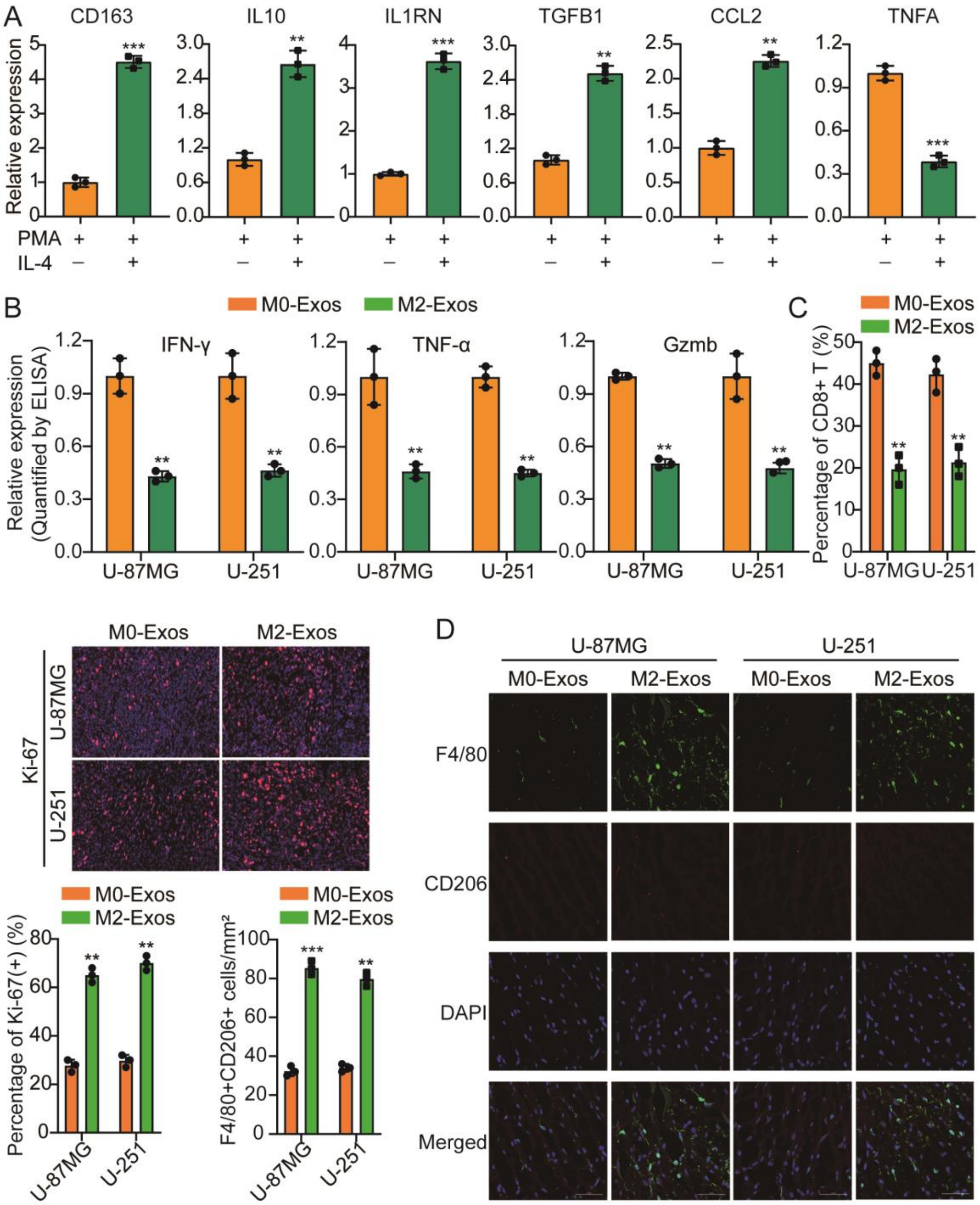

Supplementary figure 1

A. The expression level of CD163, IL10, IL1RN, TGFB1, CCL2 and TNFA was detected by qRT-PCR under different processing conditions.

B. The expression level of IFN-γ, TNF-α and Gzmb secretion was detected by ELISA under different processing conditions.

C. Typical IF picture and histogram of $CD8^+$ T and Ki-67 under different processing conditions.

D. Typical IF picture and histogram of F4/80 and CD206 under different processing

conditions.

The means ± SDs are provided (n=3). **P < 0.01 and ***P < 0.001 according to two-tailed Student's t tests or one-way ANOVA followed by Dunnett tests for multiple comparisons.

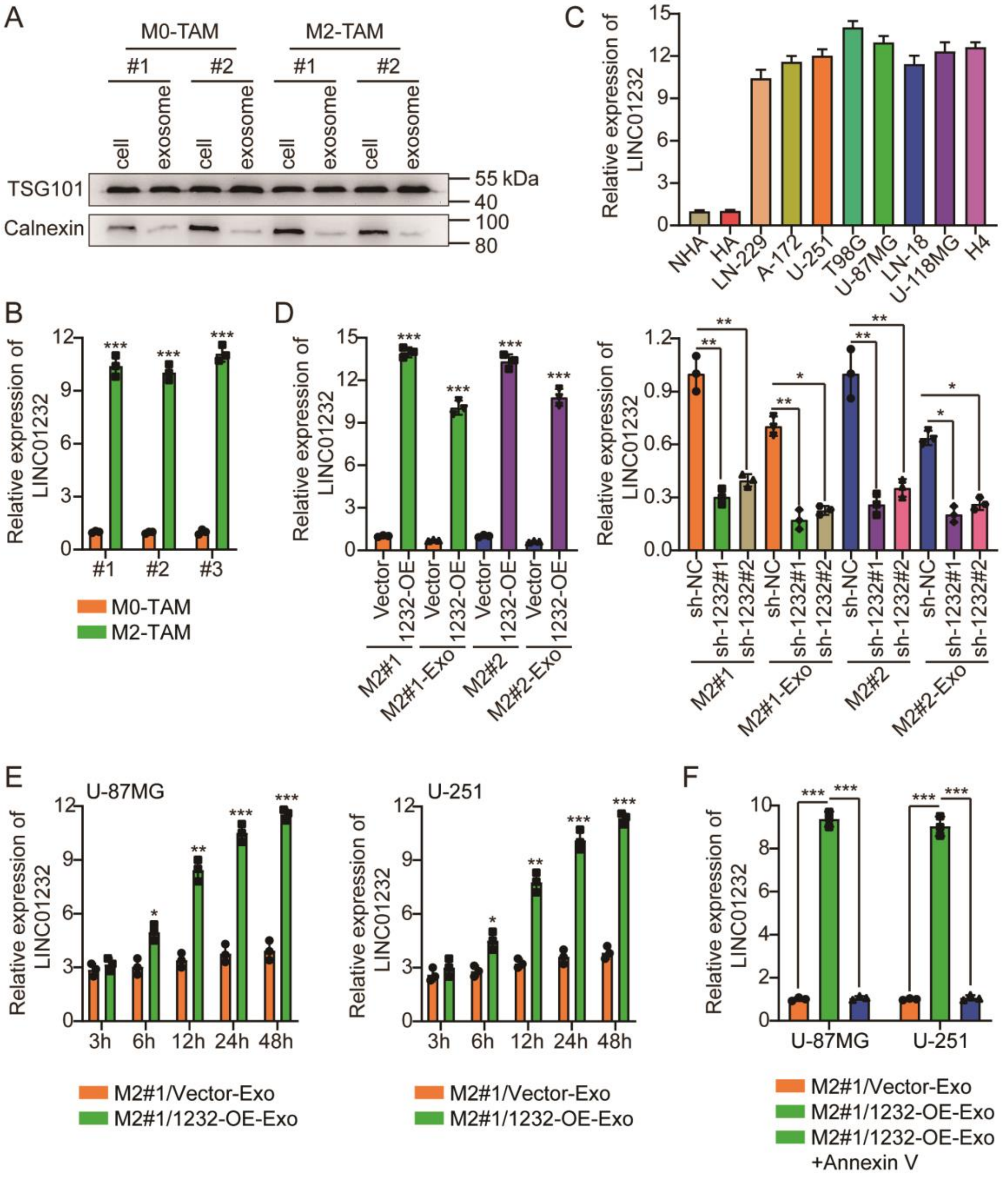

Supplementary figure 2

A. The expression level of TSG101 and Calnexin was detected by qRT-PCR under different processing conditions.

B. Measuring the levels of LINC01232 in two pairs of M0-TAM and M2-TAM.

C. The expression levels of LINC01232 were measured in normal human astrocyte cell lines (NHA, HA) and eight glioma cell lines (LN-229, A-172, U-251, T98G, U-87MG, LN-18, U-118MG, and H4) by RT-PCR.

D. The expression levels of exosomal LINC01232 were measured after overexpression or knockdown of LINC01232 in M2-TAM by RT-PCR.

E. The expression levels of LINC01232 were measured in U-87MG and U-251 incubated with exosomes derived from M2/Vector and M2/1232-OE.

F. The expression levels of LINC01232 were measured in U-87MG and U-251 incubated with exosomes derived from M2/Vector, M2/1232-OE and M2/1232-OE + Annexin V.

The means ± SDs are provided (n=3). $^{*}P < 0.05$, $^{**}P < 0.01$ and $^{***}P < 0.001$ according to two-tailed Student's t tests or one-way ANOVA followed by Dunnett tests for multiple comparisons.

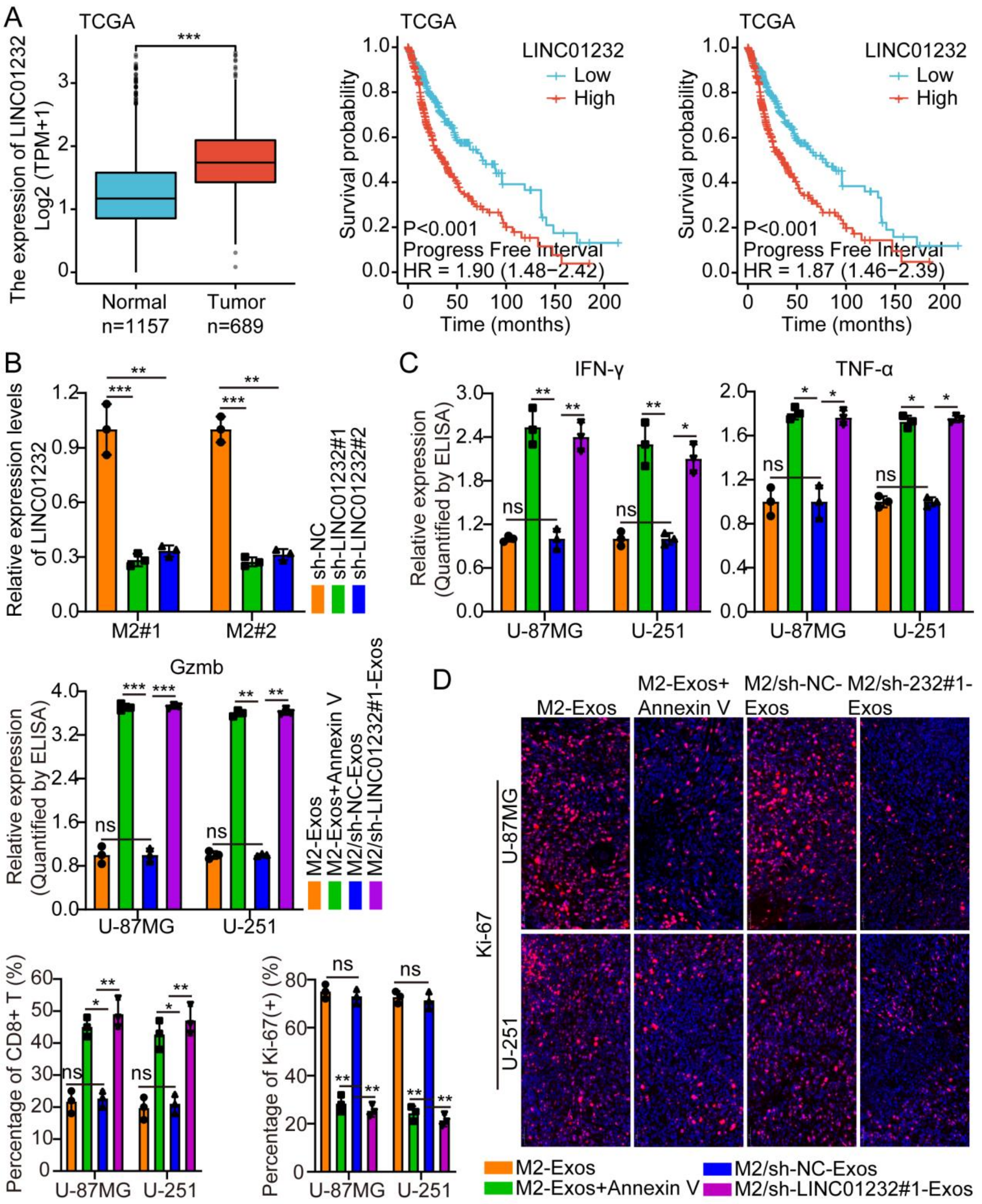

Supplementary figure 3

A. The results of the TCGA database showed that LINC01232 was highly expressed in tumor tissues, and the expression level was inversely proportional to the prognosis of patients**.**

B. Validation of knockdown efficiency of LINC01232 by qRT-PCR.

C. The expression level of IFN-γ, TNF-α and Gzmb secretion was detected by ELISA under different processing conditions.

D. Typical IF picture and histogram of CD8^{+} T and Ki-67 under different processing

conditions.

The means ± SDs are provided (n=3). *P < 0.05, **P < 0.01 and ***P < 0.001 according to two-tailed Student's t tests or one-way ANOVA followed by Dunnett tests for multiple comparisons. ns, no significant difference.

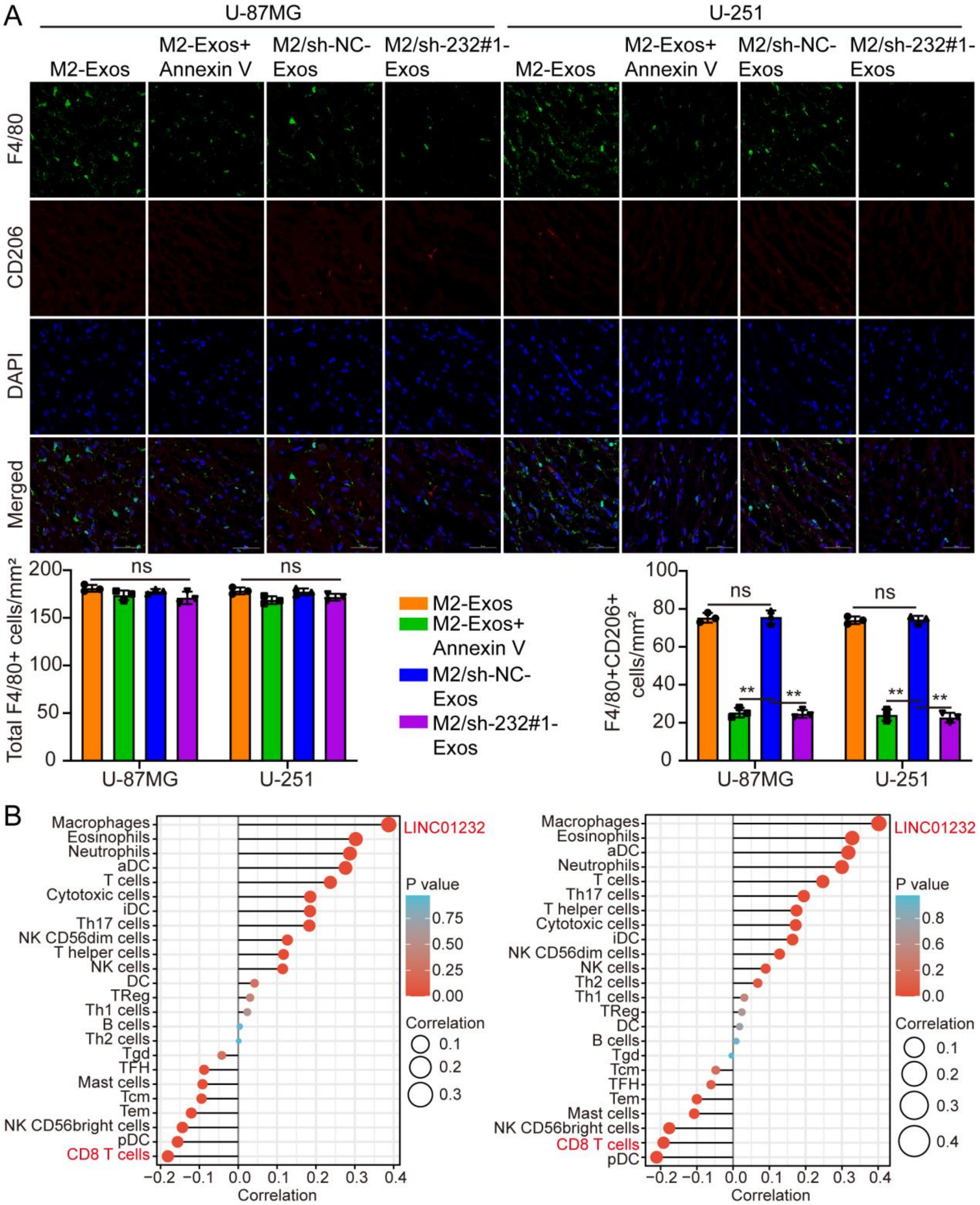

Supplementary figure 4

A. Typical IF picture and histogram of F4/80 and CD206 under different processing conditions.

B. TCGA database analysis showed that the expression level of LINC01232 was inversely proportional to the degree of infiltration of $CD8^{+}$ T cells.

The means ± SDs are provided (n=3). **P < 0.01 according to two-tailed Student's t tests or one-way ANOVA followed by Dunnett tests for multiple comparisons. ns, no significant difference.

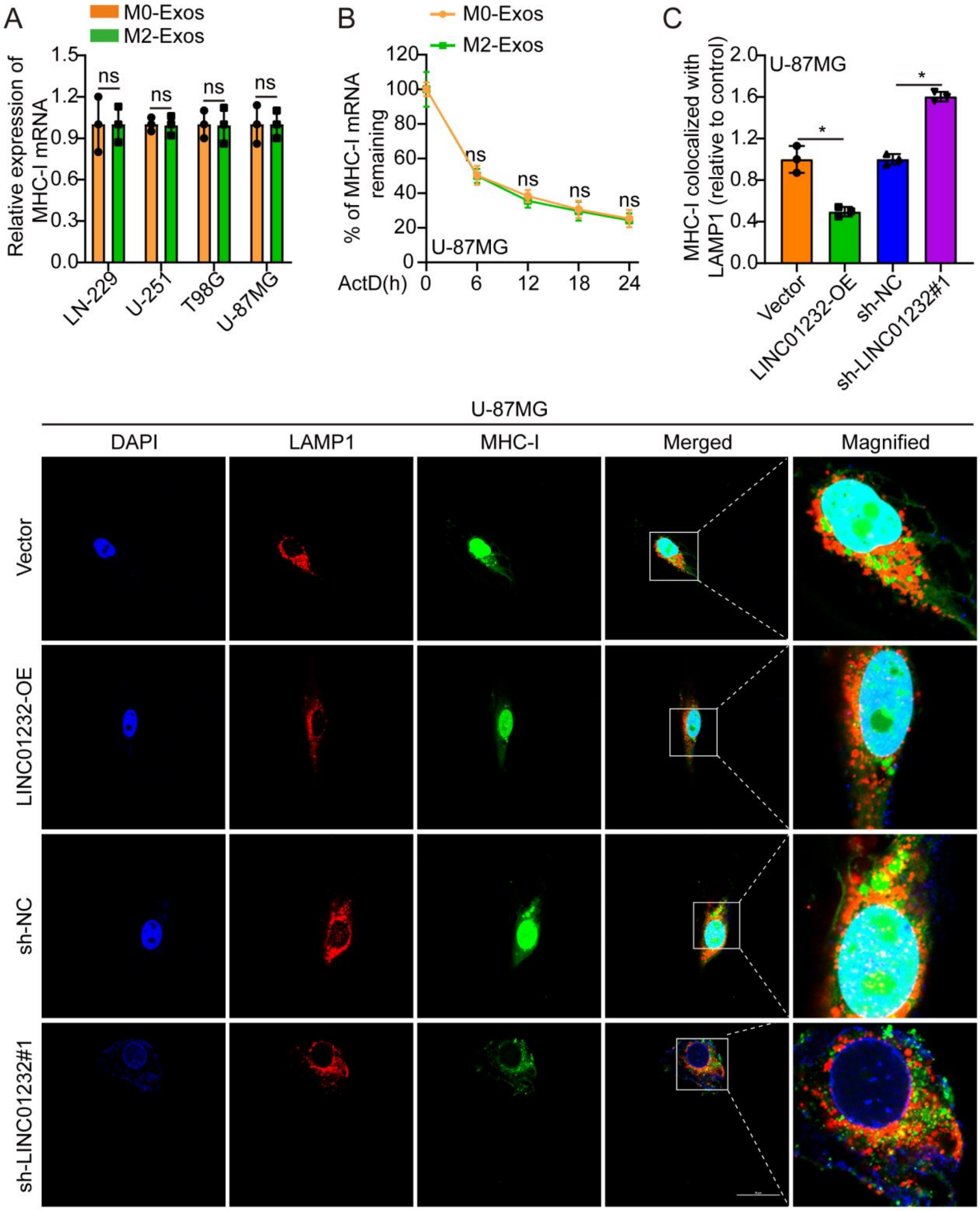

Supplementary figure 5

A. The expression level of MHC-I was detected by qRT-PCR under different

processing conditions.

B. Typical MHC-I mRNA degradation curve under different processing conditions.

C. Representative confocal images showing the expression levels of MHC-I in lysosomes of U-87MG cells treated with Vector, LINC01232-OE, sh-NC and sh-LINC01232#1. Red represents LAMP1; green represents MHC-I; yellow represents the fusion of red and green.

The means ± SDs are provided (n=3). *$P < 0.05$ according to two-tailed Student's t tests or one-way ANOVA followed by Dunnett tests for multiple comparisons. ns, no significant difference.

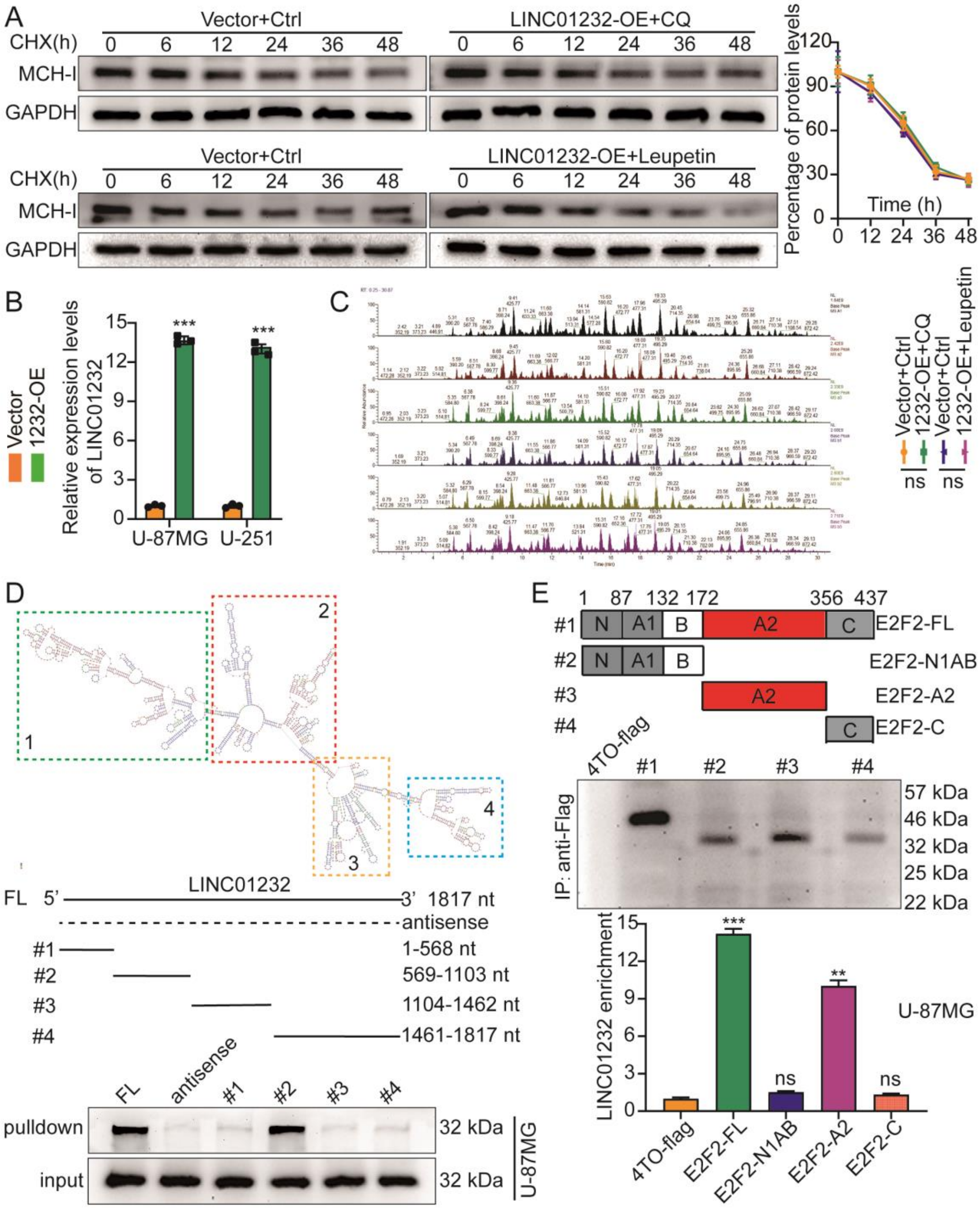

Supplementary figure 6

A. U-87MG cells expressing either Vector+Ctrl or LINC01232-OE+CQ and U-251MG cells expressing either Vector+Ctrl or LINC01232-OE+Leupetin were treated with CHX for corresponding time. Cell lysates were measured by WB with corresponding antibodies. Using ImageJ to analyze the protein band intensity. Right: Typical MHC-I protein degradation curve.

B. Validation of overexpression efficiency of LINC01232 by qRT-PCR.

C. The mass spectrometry data.

D. Immunoblotting detection of the E2F2 proteins in U-87MG cells as extracted via transcribed biotinylated RNAs in vitro of different constructs of LINC01232 or its antisense sequences (negative control).

E. RIP assays were conducted with anti-Flag antibodies in HEK293T cells transfected with Flag-tagged E2F2 vector or its deletion mutants (4TO-Flag vector used as negative control). qRT-PCR was used to detect the enrichment of LINC01232. Western blotting was used to measure the expression levels of Flag-tagged E2F2 or its deletion mutants.

The means ± SDs are provided (n=3). $^{**}P < 0.01$ and $^{***}P < 0.001$ according to two-tailed Student's t tests or one-way ANOVA followed by Dunnett tests for multiple comparisons. ns, no significant difference.

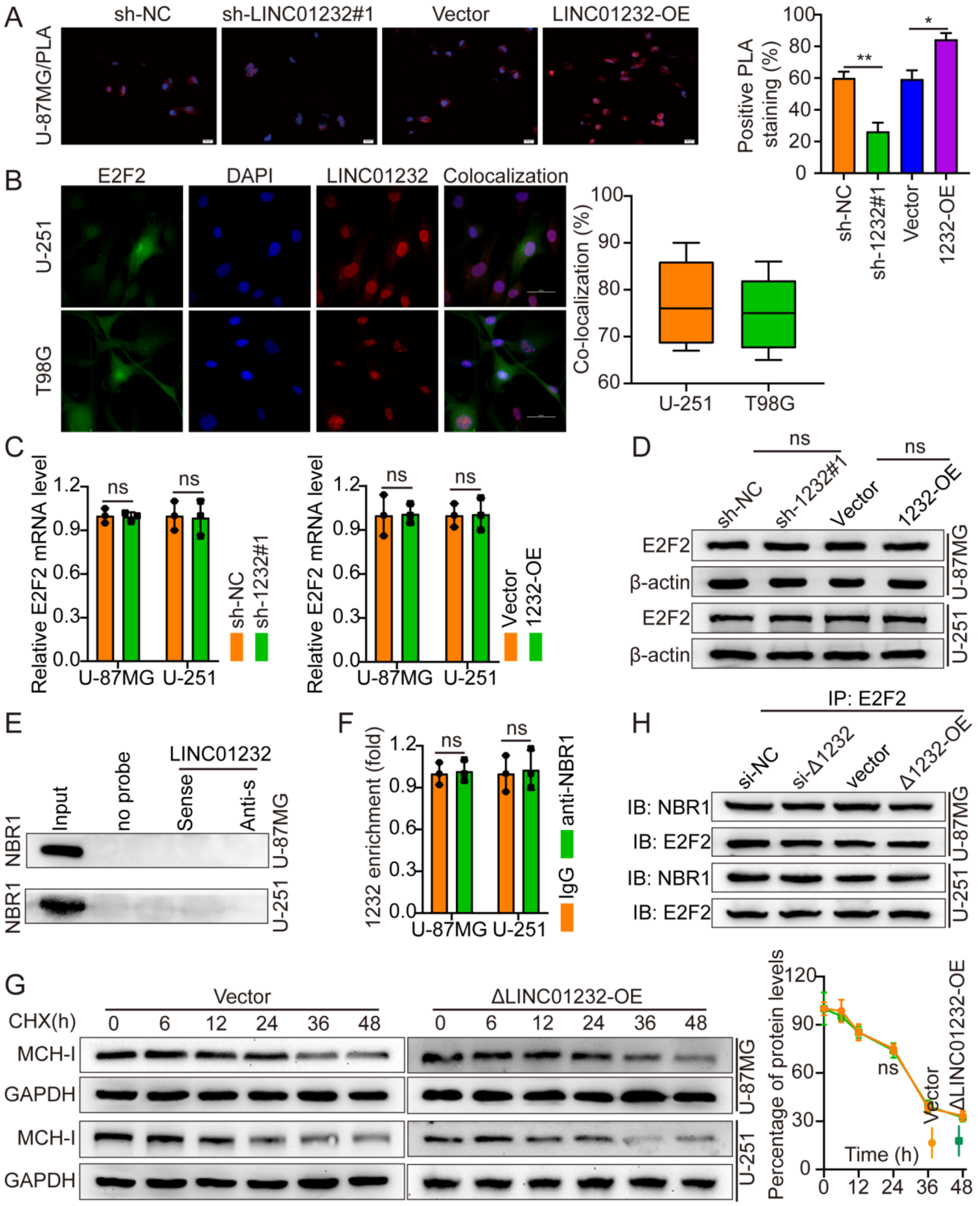

Supplementary figure 7

A. PLA on U-87MG cells measured the interaction between E2F2 and NBR1 in the LINC01232-knockdown, LINC01232-overexpressing, and corresponding control groups. Positive PLA signal showed that the E2F2–NBR1 complexes were clustered in red and the nuclei we counterstained in blue. Right: Typical histogram.

B. Fish-IF assay to prove LINC01232 directly bind to E2F2.

C. The expression level of E2F2 mRNA was detected by qRT-PCR under different conditions.

D. The expression level of E2F2 protein was detected by WB under different conditions.

E. Western blotting analysis of the proteins obtained from the LINC01232 pull-down assay with anti-NBR1 antibodies.

F. RIP assays with anti-NBR1 antibodies indicated that NBR1 did not interact with LINC01232 in U-251MG and U-87MG cells.

G. U-87MG and U-251 cells expressing either Vector or ΔLINC01232-OE were treated with CHX for corresponding time. Cell lysates were measured by WB with corresponding antibodies. Using ImageJ to analyze the protein band intensity. Right: Typical MHC-I protein degradation curve.

H. Co-IP assays were conducted to determine the E2F2–NBR1 interaction in the LINC01232 knockdown, LINC01232 overexpressing, and control groups.

The means ± SDs are provided (n=3). *$P < 0.05$ and **$P < 0.01$ according to two-tailed Student's t tests or one-way ANOVA followed by Dunnett tests for multiple comparisons. ns, no significant difference.

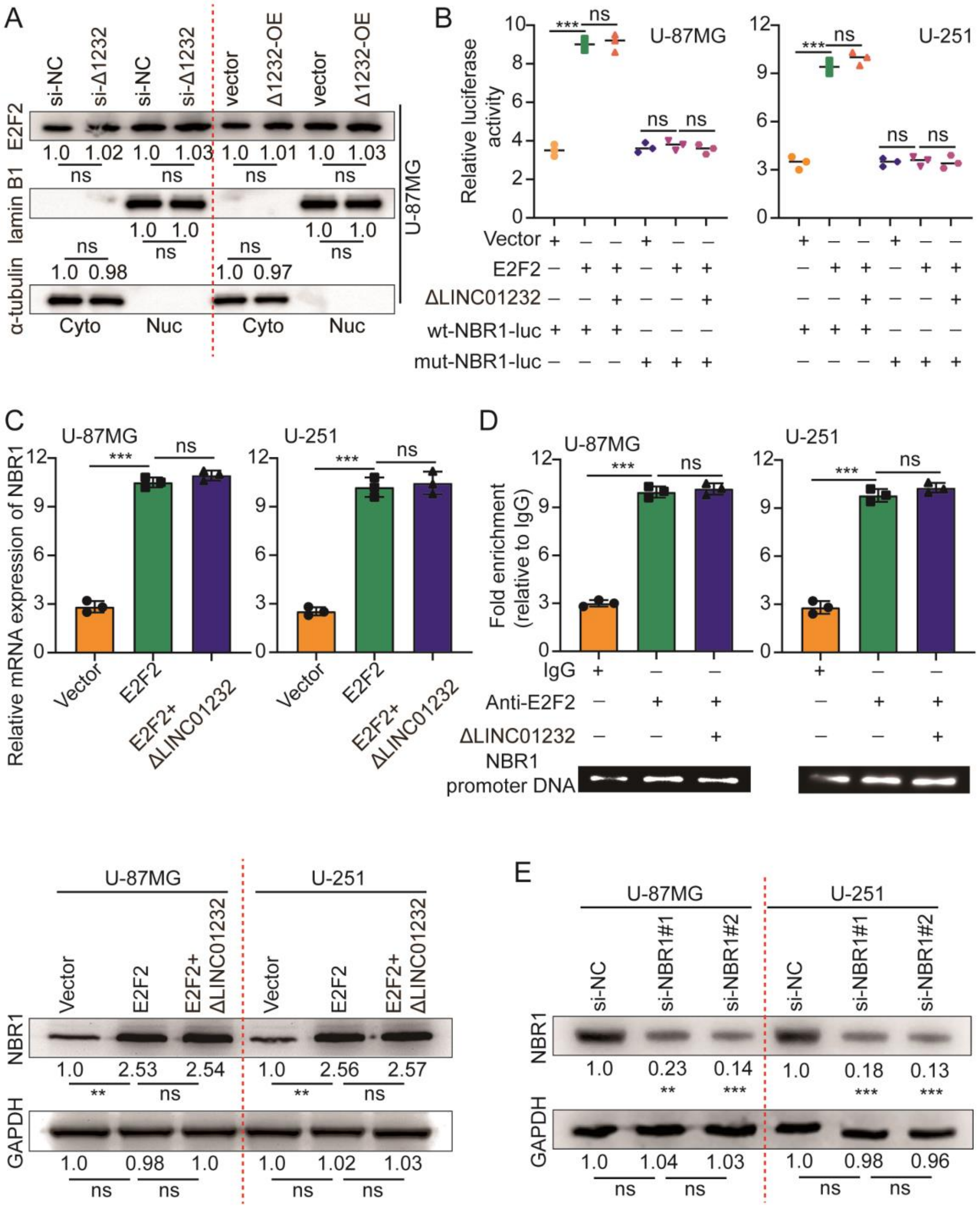

Supplementary figure 8

A. Nuclear and cytosolic lysates were separated from U-87MG with knockdown or overexpression of ΔLINC01232, followed by WB analysis with corresponding antibodies

B. Luciferase activity was assayed in U-87MG and U-251 cells transfected with luciferase vectors (wild type or mutant type) and meantime co-transfected with expression plasmids (empty vectors, E2F2 expression plasmids, or ΔLINC01232 expression plasmids).

C. The level of NBR1 under ectopic expression of E2F2 or ΔLINC01232 was detected by qRT-PCR and western blotting.

D. ChIP experiments of E2F2 (IgG as an internal control) were performed, and the co-precipitated DNA was subjected to PCR amplification with primers specific to NBR1 promoter region.

E. Validation of knockdown efficiency of NBR1 between U-87MG and U-251 by WB.

The means ± SDs are provided (n=3). *P < 0.05 and **P < 0.01 according to two-tailed Student's t tests or one-way ANOVA followed by Dunnett tests for multiple comparisons. ns, no significant difference.

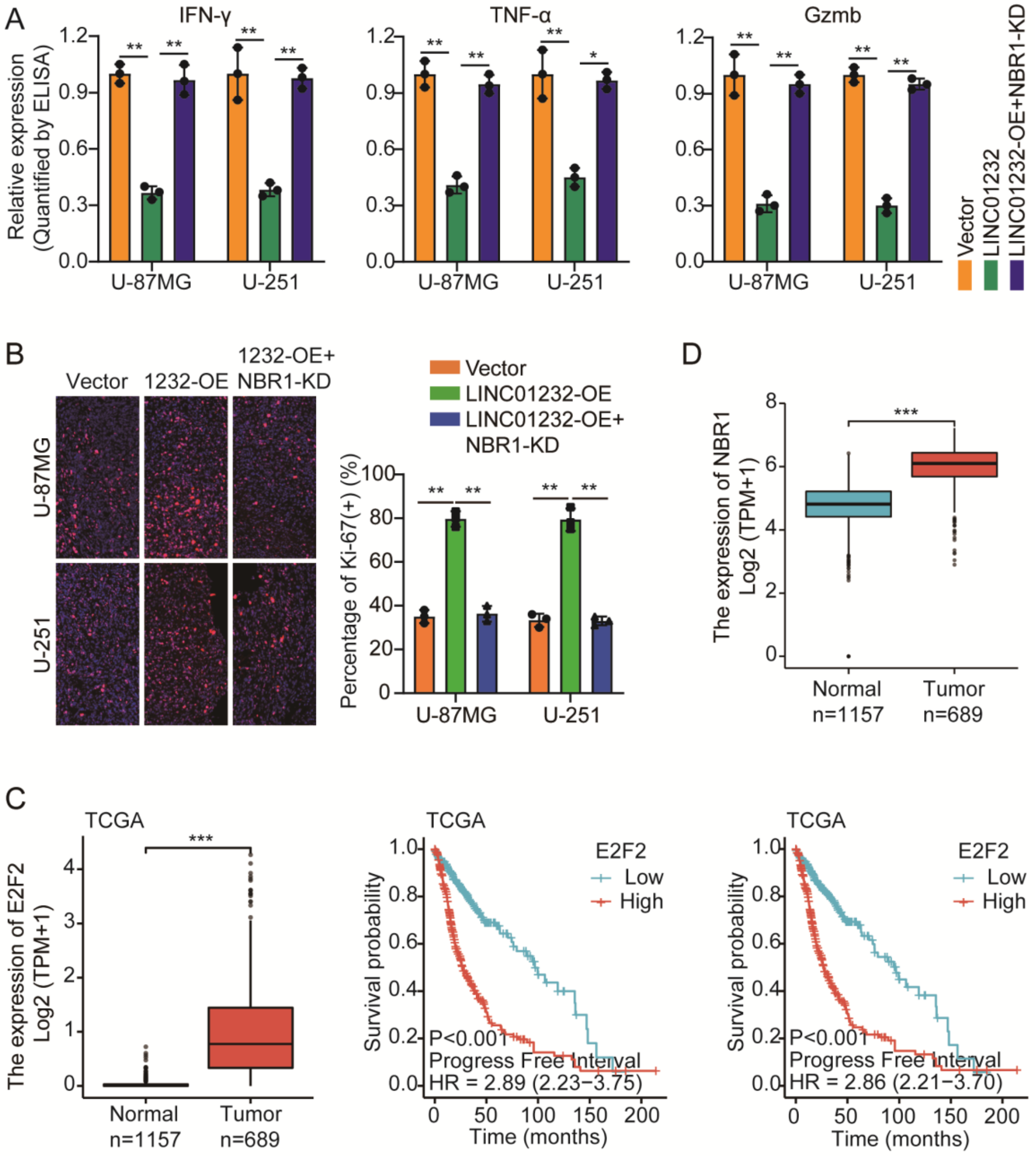

Supplementary figure 9

A. The expression level of IFN-γ, TNF-α and Gzmb secretion was detected by ELISA under different processing conditions.

B. Typical IF picture and histogram of Ki-67 under different processing conditions.

C. The results of the TCGA database showed that E2F2 was highly expressed in tumor tissues, and the expression level was inversely proportional to the prognosis of patients.

D. The results of the TCGA database showed that NBR1 was highly expressed in tumor tissues.

The means ± SDs are provided (n=3). *P < 0.05, **P < 0.01 and ***P < 0.001 according to two-tailed Student's t tests or one-way ANOVA followed by Dunnett tests for multiple comparisons.

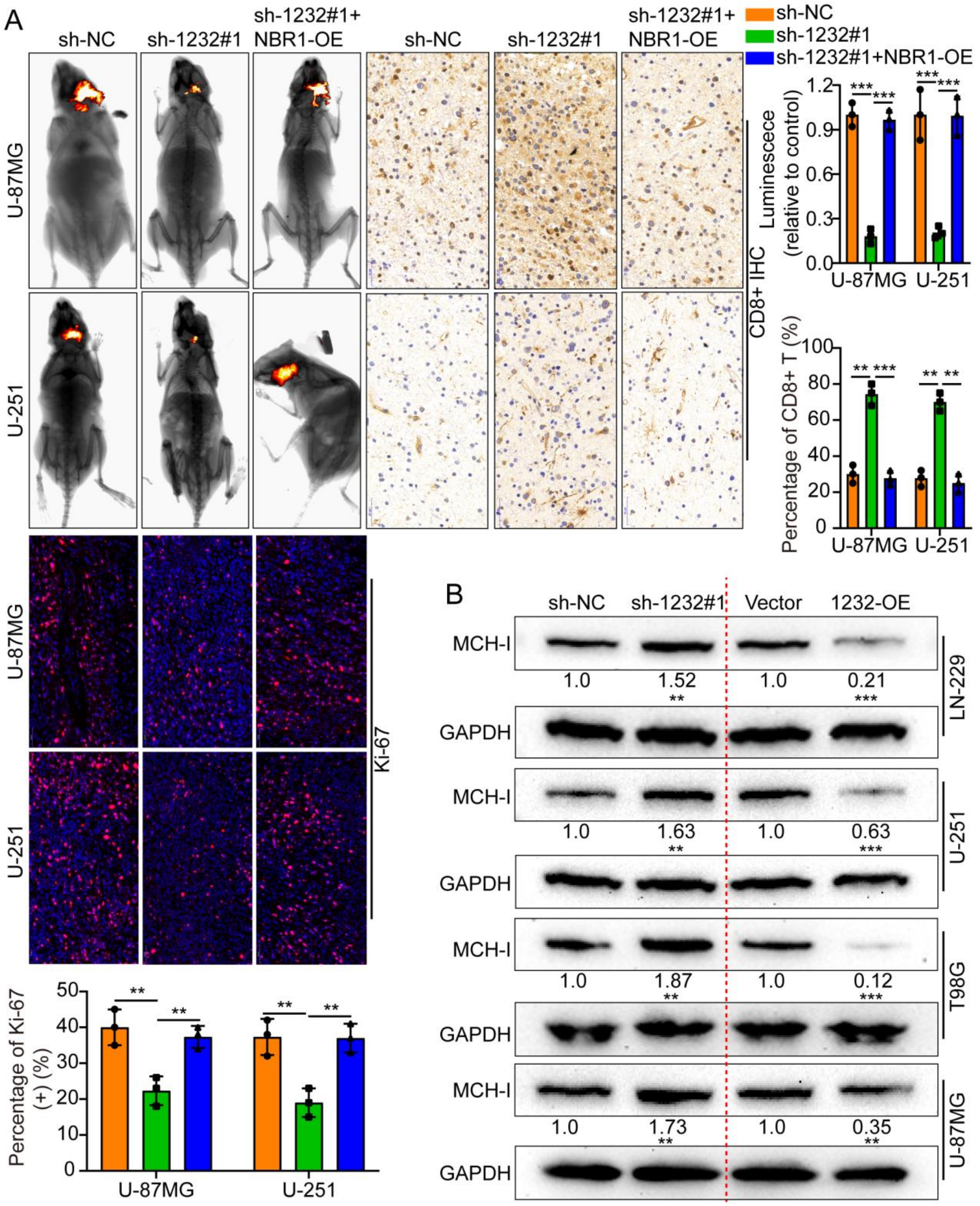

Supplementary figure 10

A. Typical animal in vivo imaging pictures, Ki-67 IF pictures and $CD8^+$ IHC pictures in different groups. Scale bar, 50um.

B. The expression level of MHC-I protein was detected by WB between sh-NC,

sh-LINC01232#1, Vector and LINC01232-OE in different tumor cells line.

The means ± SDs are provided (n=3). **P < 0.01 and ***P < 0.001 according to two-tailed Student's t tests or one-way ANOVA followed by Dunnett tests for multiple comparisons.

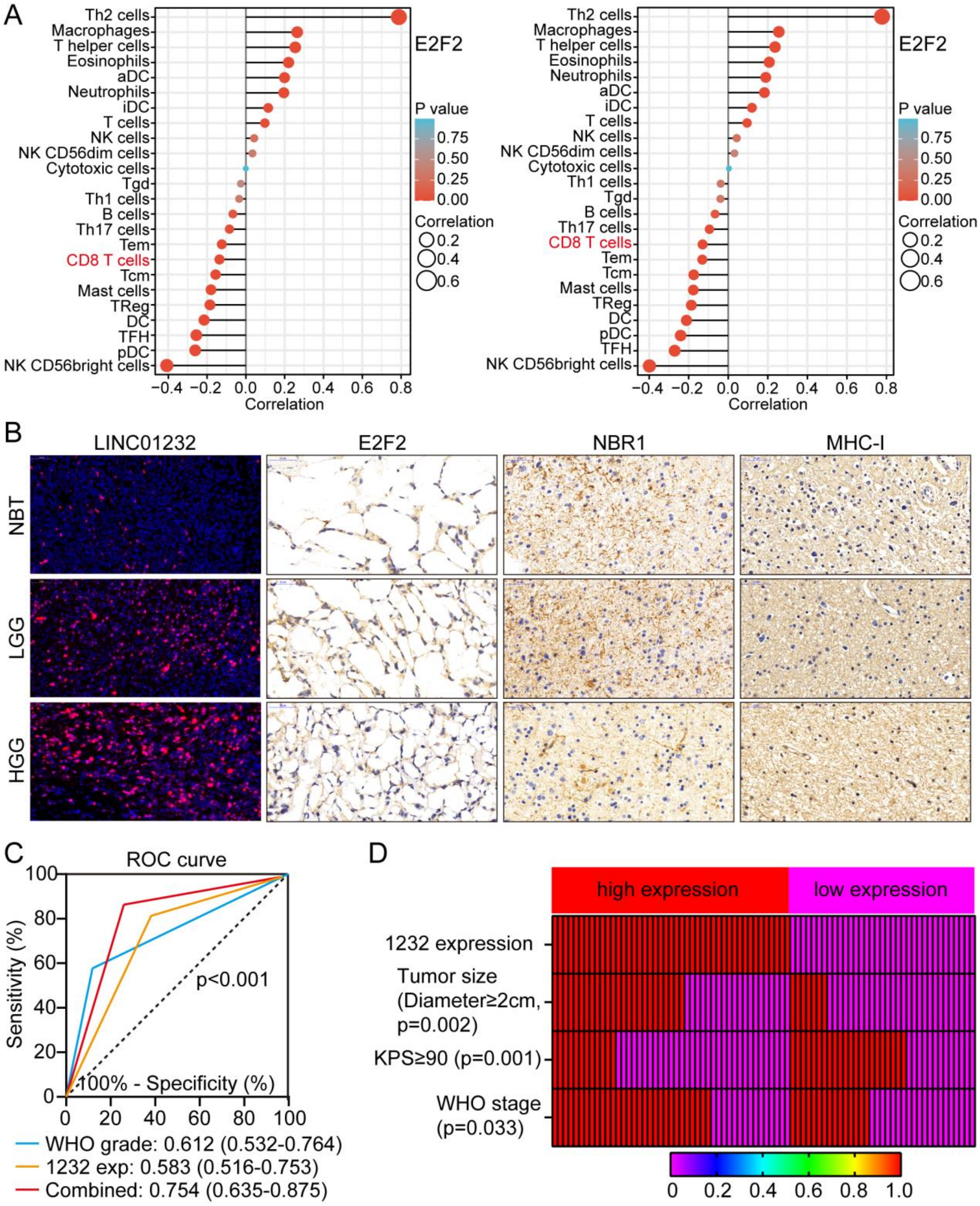

Supplementary figure 11

A. TCGA database analysis showed that the expression level of E2F2 was inversely proportional to the degree of infiltration of $CD8^+$ T cells.

B. Spearman correlation analysis between LINC01232 expression levels and E2F2,

NBR1 and MHC-I expression levels in glioma tissues. Pearson's correlation coefficient (r) and P-value as the picture showed; P-value was from Spearman's test. Scale bar represents 50 μm.

C. ROC analysis of LINC01232-based, WHO-based and the combination model in predicting clinical outcome.

D. The heatmap illustrates the association of different clinical characters with LINC01232 high and low-expression tumors.

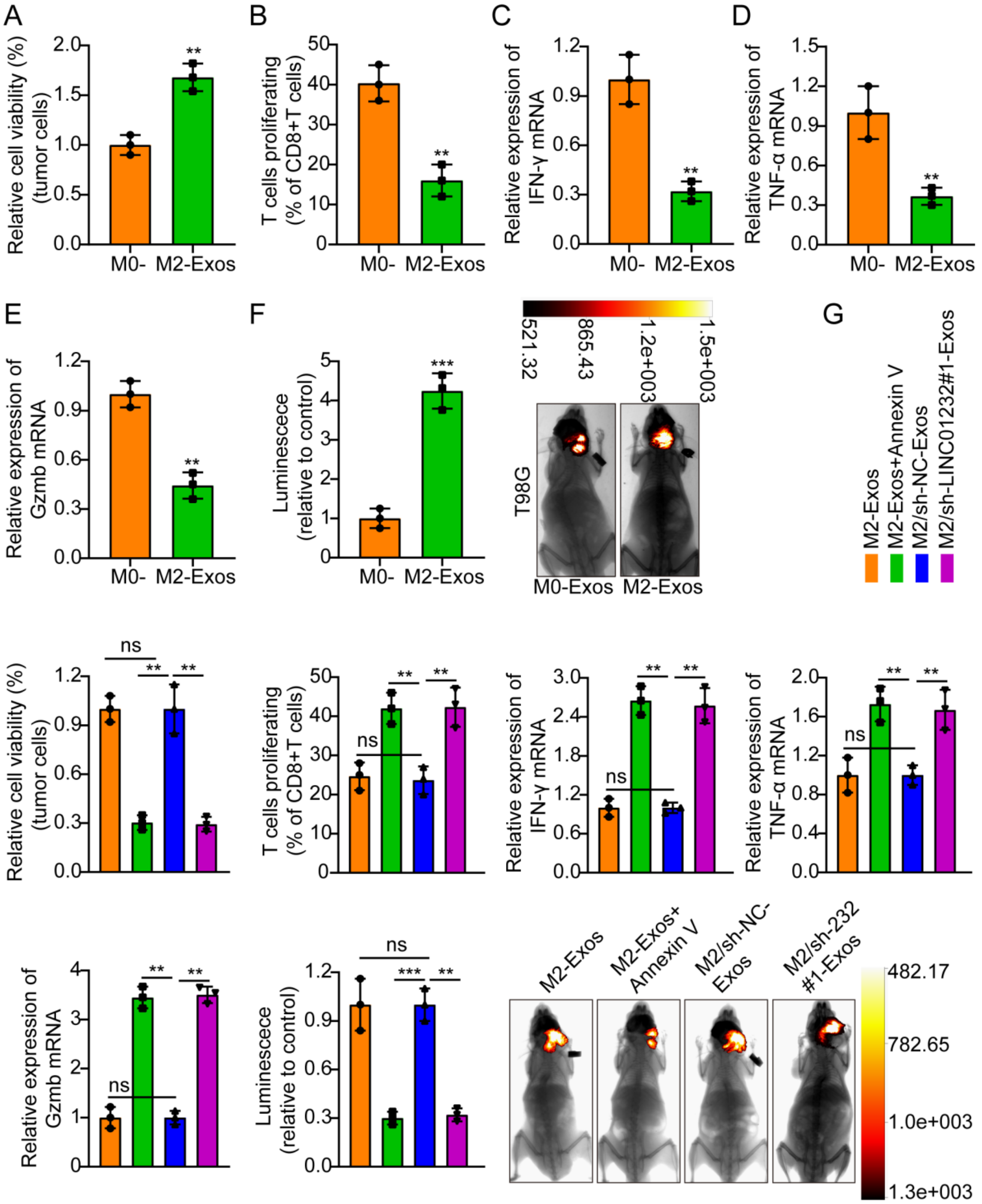

Supplementary figure 12

A. M2-Exos significantly inhibited T cell-mediated tumor cell killing compared with M0-Eoxs.

B-E. Flow cytometry and real-time quantitative PCR results indicated that $CD8^{+}$ T cells co-cultured with M2-Exos-glioma cells showed lower proliferation and expression of IFN-γ, TNF-α and Gzmb.

F. Typical animal in vivo imaging pictures, typical column chart in different groups. Scale bar, 50um.

G. M2-Exos significantly inhibited T cell-mediated tumor cell killing compared with M0-Eoxs. However, after adding an exosome inhibitor Annexin V or knocking down LINC01232, the above phenomenon was restored. Flow cytometry and real-time quantitative PCR results indicated that $CD8^{+}$ T cells co-cultured with M2-Exos-glioma cells showed lower proliferation and expression of IFN-γ, TNF-α and Gzmb. However, after adding an exosome inhibitor Annexin V or knocking down LINC01232, the above phenomenon was restored. Typical animal in vivo imaging pictures, typical column chart in different groups. Scale bar, 50um.

The means ± SDs are provided (n=3). **P < 0.01 and ***P < 0.001 according to two-tailed Student's t tests or one-way ANOVA followed by Dunnett tests for multiple comparisons. ns, no significant difference.

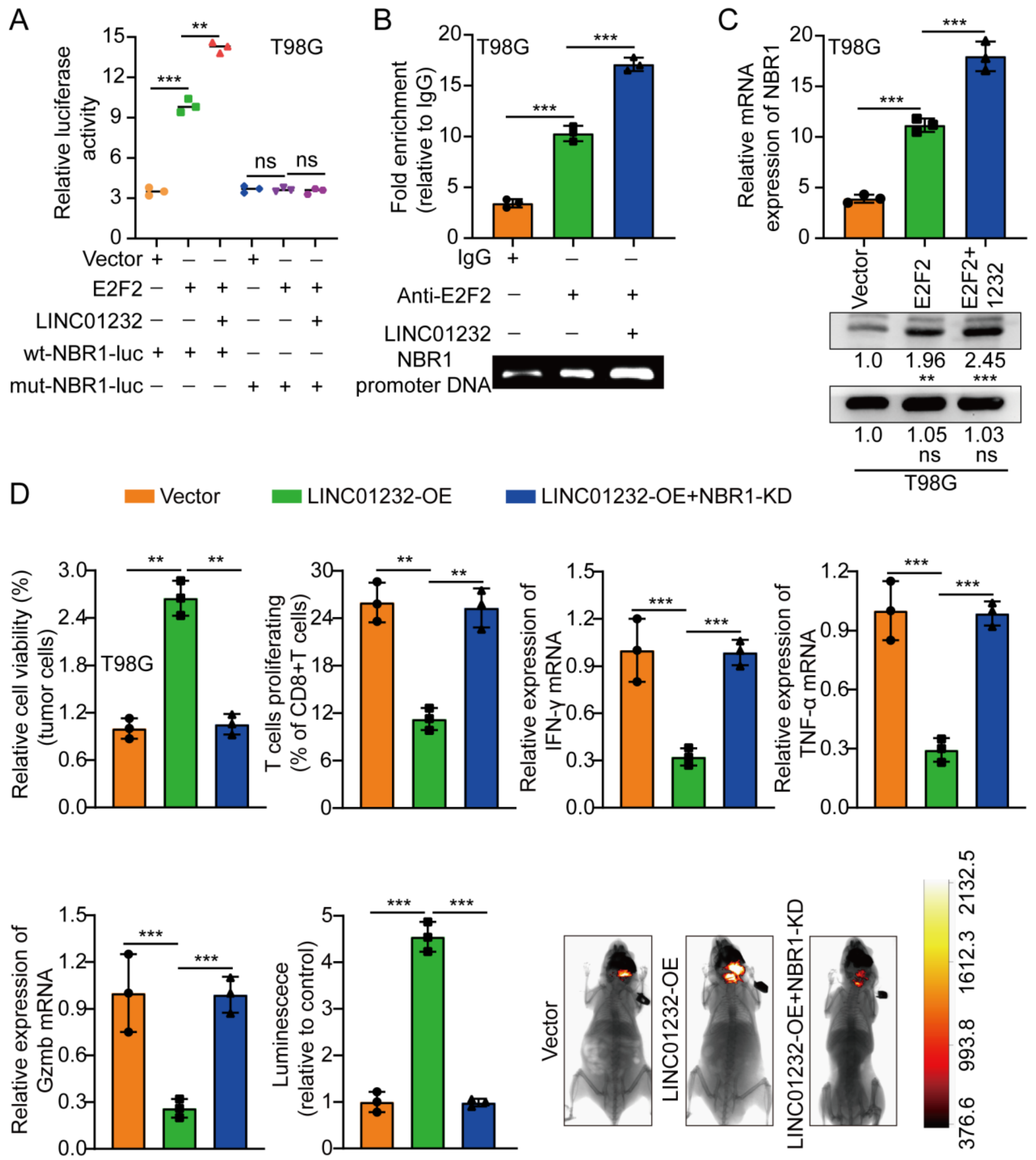

Supplementary figure 13

A. Luciferase activity was assayed in U-87MG and U-251 cells transfected with luciferase vectors (wild type or mutant type) and meantime co-transfected with expression plasmids (empty vectors, E2F2 expression plasmids, or LINC01232 expression plasmids).

B. ChIP experiments of E2F2 (IgG as an internal control) were performed, and the co-precipitated DNA was subjected to PCR amplification with primers specific to NBR1 promoter region.

C. The level of NBR1 under ectopic expression of E2F2 or LINC01232 was detected

by qRT-PCR and western blotting.

D. LINC01232-OE/U-87MG/T98G cells significantly inhibited T cell-mediated tumor cell killing compared with Vector; however, knocking down NBR1 while overexpressing LINC01232 could rescue the above phenomenon. Flow cytometry and real-time quantitative PCR results indicated that $CD8^+$ T cells co-cultured with LINC01232-OE-glioma cells showed lower proliferation and expression of IFN-γ, TNF-α and Gzmb; however, knocking down NBR1 while overexpressing LINC01232 could rescue the above phenomenon. Typical animal in vivo imaging pictures and typical column chart in different groups. Scale bar, 50um.

The means ± SDs are provided (n=3). $^{**}P < 0.01$ and $^{***}P < 0.001$ according to two-tailed Student's t tests or one-way ANOVA followed by Dunnett tests for multiple comparisons. ns, no significant difference.

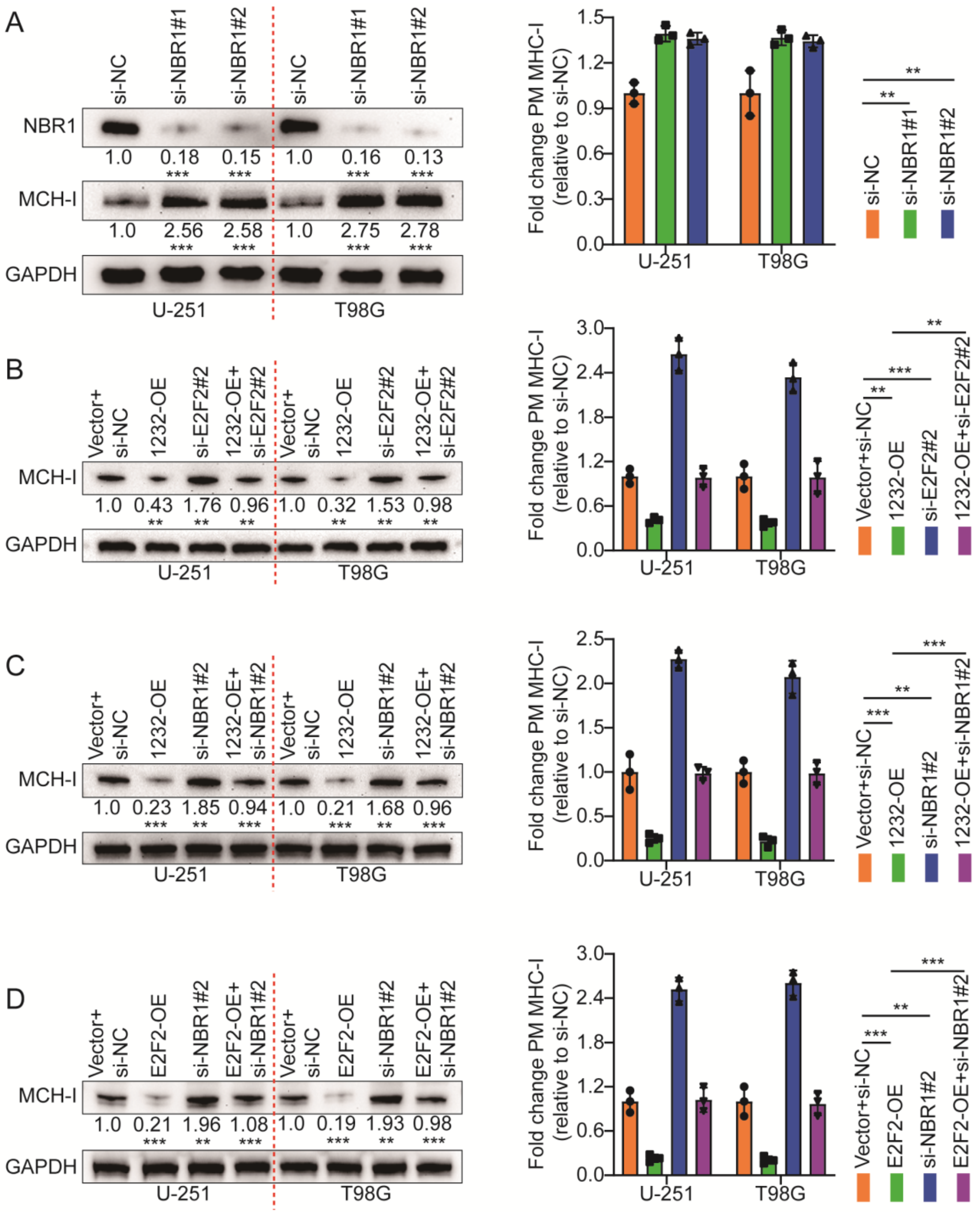

Supplementary figure 14

A. Knockdown of NBR1 in U-251 and T98G, respectively, and the expression level of MHC-I was detected by WB. Flow cytometry-based quantification of plasma membrane MHC-I (n = 3 replicates from three independent experiments) after NBR1 knockdown.

B-D. The expression level of MHC-I was detected by WB under different groups. Flow cytometry-based quantification of plasma membrane MHC-I (n = 3 replicates from three independent experiments) under different groups.

The means ± SDs are provided (n=3). **P < 0.01 and ***P < 0.001 according to two-tailed Student's t tests or one-way ANOVA followed by Dunnett tests for multiple comparisons. ns, no significant difference.

References

[1] a) J. Li, T. Liao, H. Liu, H. Yuan, T. Ouyang, J. Wang, S. Chai, J. Li, J. Chen, X. Li, H. Zhao, N. Xiong, *Cancer Res* 2021, *81* (1), 114, https://doi.org/10.1158/0008-5472.CAN-20-2270; b) J. Li, H. Yuan, H. Xu, H. Zhao, N. Xiong, *Mol Cancer Res* 2020, *18* (8), 1218, https://doi.org/10.1158/1541-7786.MCR-19-0725;

[2] G. T. Consortium, *Nat Genet* 2013, *45* (6), 580, https://doi.org/10.1038/ng.2653.

[3] J. Vivian, A. A. Rao, F. A. Nothaft, C. Ketchum, J. Armstrong, A. Novak, J. Pfeil, J. Narkizian, A. D. Deran, A. Musselman-Brown, H. Schmidt, P. Amstutz, B. Craft, M. Goldman, K. Rosenbloom, M. Cline, B. O'Connor, M. Hanna, C. Birger, W. J. Kent, D. A. Patterson, A. D. Joseph, J. Zhu, S. Zaranek, G. Getz, D. Haussler, B. Paten, *Nat Biotechnol* 2017, *35* (4), 314, https://doi.org/10.1038/nbt.3772.

[4] J. Liu, T. Lichtenberg, K. A. Hoadley, L. M. Poisson, A. J. Lazar, A. D. Cherniack, A. J. Kovatich, C. C. Benz, D. A. Levine, A. V. Lee, L. Omberg, D. M. Wolf, C. D. Shriver, V. Thorsson, N. Cancer Genome Atlas Research, H. Hu, *Cell* 2018, *173* (2), 400, https://doi.org/10.1016/j.cell.2018.02.052.
